# Supplementary material for: Monocyte depletion enhances neutrophil influx and proneural to mesenchymal transition in glioblastoma
Source: Nat Commun. 2023 Apr 3;14:1839. doi: 10.1038/s41467-023-37361-8 (PMC10070461; doi:10.1038/s41467-023-37361-8)
Supplement: Supplementary file 1 — Supplementary Information [file 41467_2023_37361_MOESM1_ESM.pdf]

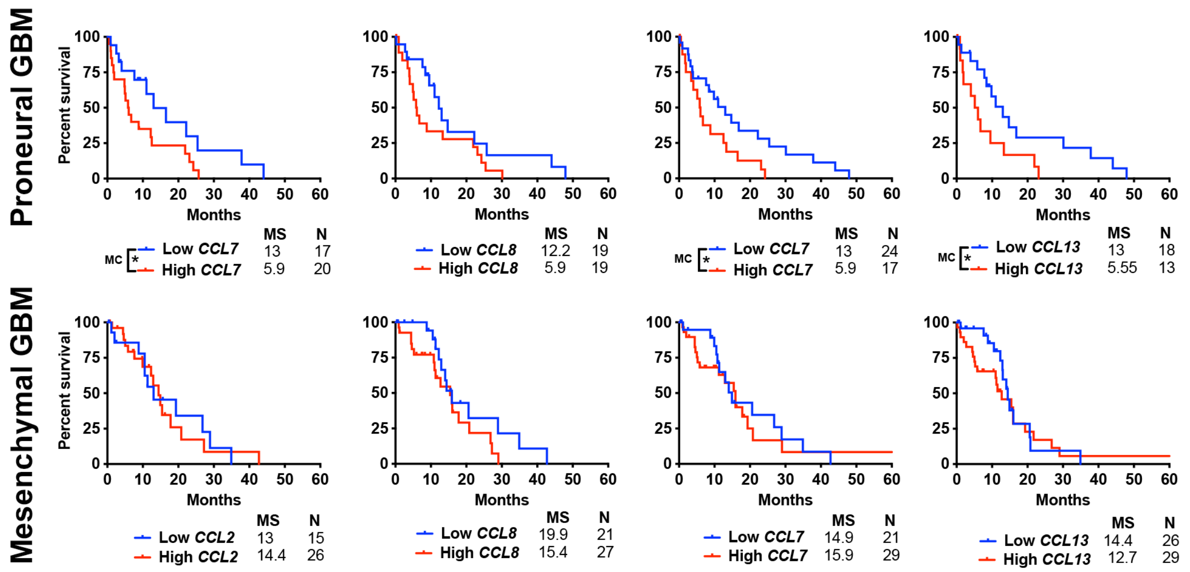

Supplementary Figure 1. High expression of MCPs except *CCL8* correlated with inferior survival of PN GBM patient; in contrast, MCP expressions do not correlate with survival of MES GBM patients. (A) Kaplan-Meier survival curves of PN and (B) MES GBM patients based on high and low expression of MCPs. High and Low were defined as +/-0.5 SD from the mean of corresponding subtype (PN = 54, MES =77). MS = median survival by months and N = number of patients. Log-rank test (MC), * $P < 0.05$. Source data are provided as a Source Data file.

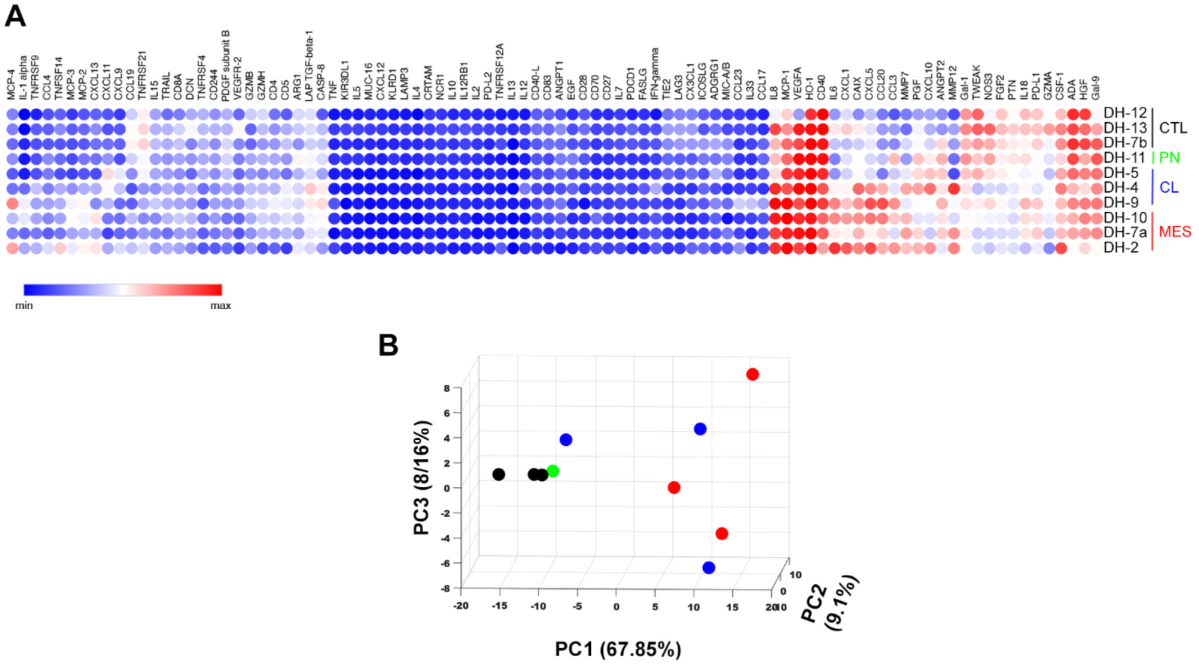

Supplementary Figure 2. Illustration of Olink multiplex proteomics with 96 immune protein targets in human control brain and GBM subsets. (A) Heatmap showing normalized expression score of the analytes. (B) Principal component analysis of the results. CTL (black) = control, PN (green)= proneural, CL (blue) = classical, MES (red) = mesenchymal. Source data are provided as a Source Data file.

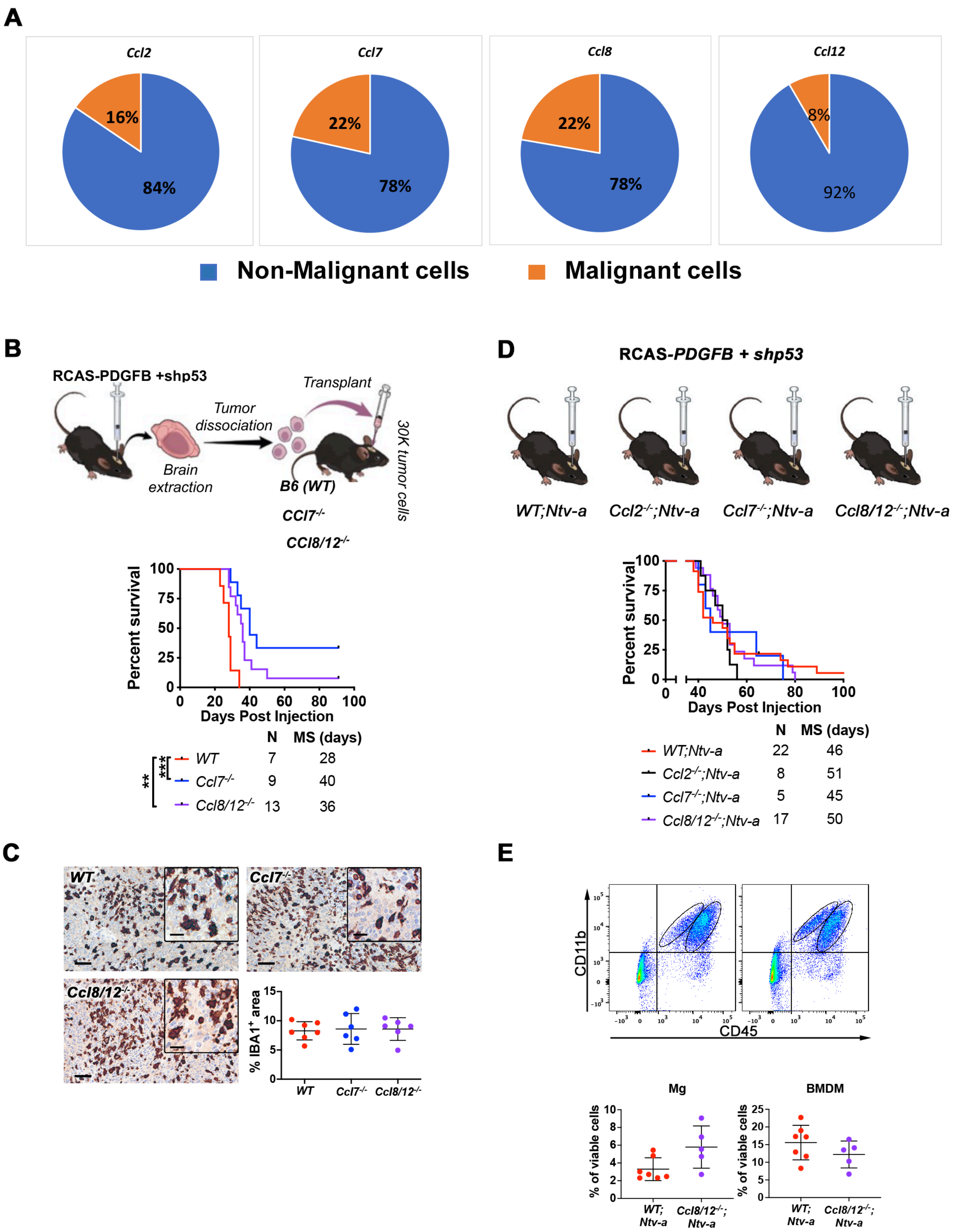

Supplementary Figure 3. ***MCPs*** **are expressed by both non-malignant and malignant cells. Loss of** ***Ccl7*** **or** ***Ccl8/12*** **in TME but not tumor cells result in extended survival of GBM-bearing mice.** (A) Pie charts of quantification of CCL expression between non-malignant and malignant cells based on scRNA-seq data from *PDGFB*-driven GBM in *WT; Ntv-a* mice. (B) Schematic illustration of orthotopic transplant of primary *PDGFB*-driven *WT* tumors into *Ccl7*$^{-/-}$

and *Ccl8/12*$^{-/-}$ (CCL7 or CCL8/12 are lost only in TME), and *WT* recipient animals and their corresponding Kaplan-Meier survival curves. Log-rank test. P = 0.0004 (*WT* vs. *Ccl8/12*$^{-/-}$) and 0.0014 (*WT* vs. *Ccl7*$^{-/-}$), respectively. (C) Representative images and quantification of immunohistochemical staining of IBA1. N = 7 (WT) and 6 (*Ccl7*$^{-/-}$ and *Ccl8/12*$^{-/-}$). Data are presented as mean +/- SD. (D) Schematic illustration of injections and Kaplan-Meier survival curves of *PDGFB*-driven tumors generated in *WT; Ntv-a*, *Ccl2*$^{-/-}$*; Ntv-a, Ccl7*$^{-/-}$*; Ntv-a* and *Ccl8/12*$^{-/-}$*; Ntv-a* mice. (E) Dot plots of flow cytometry analysis of BMDM and Mg in tumors at humane endpoint. N = 7 (*WT; Ntv-a)* and 5 *(Ccl8/12*$^{-/-}$*; Ntv-a* mice). Data are presented as mean +/- SD. Two-sided Student's *t*-test. ** $P < 0.01$, *** $P < 0.001$. Scale bar = 50 µm, scale bar in inset = 20 µm. Source data are provided as a Source Data file.

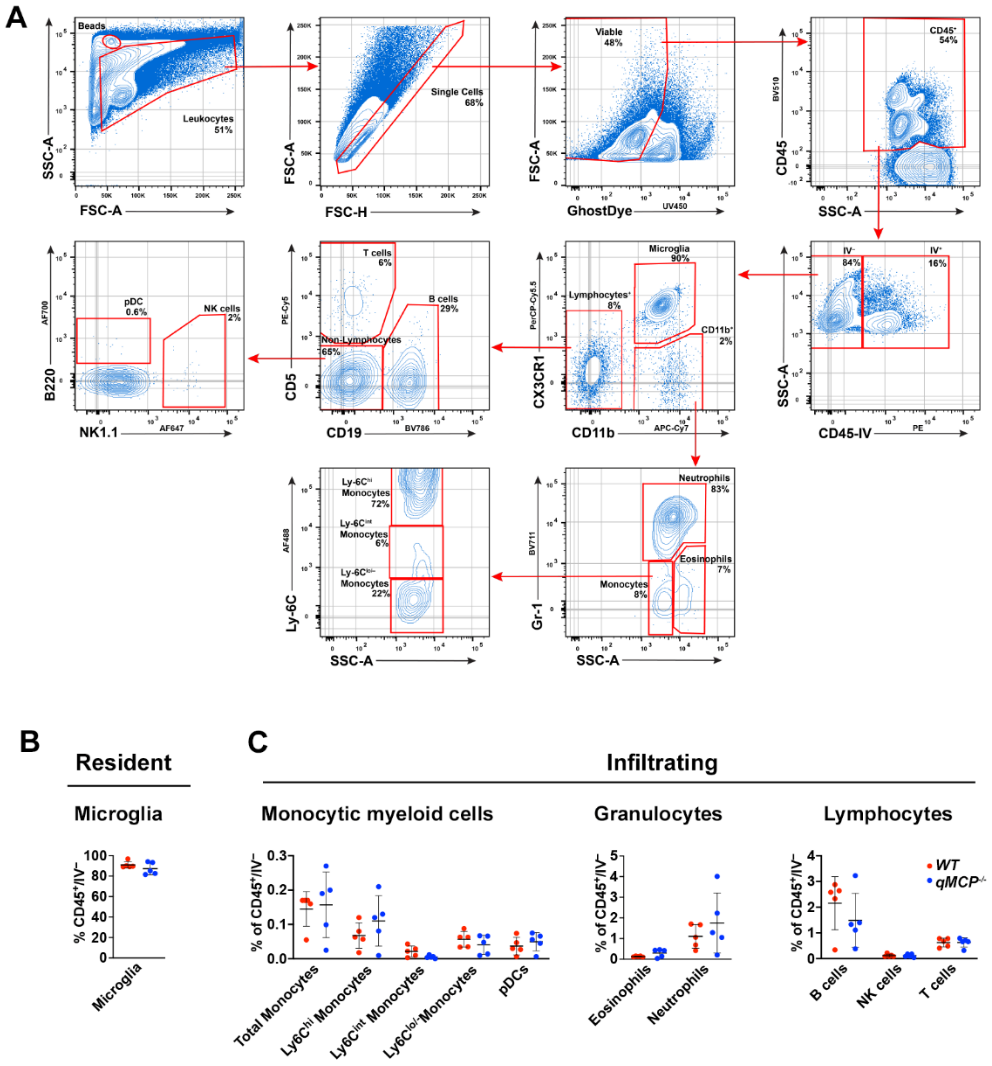

Supplementary Figure 4. Flow cytometric analysis of brain resident and infiltrating immune cells in healthy adult *WT* and *qMCP*$^{-/-}$ mice. (A) Gating strategy identifying subsets of immune cells from of *WT* and *qMCP*$^{-/-}$ mice. (B) Quantification of resident MG. N = 5 for both *WT* and *qMCP*$^{-/-}$ mice. Data are presented as mean +/- SD. (C) Quantification of brain infiltrating immune cells. Each dot represents an independent mouse. Data are presented as mean +/- SD. N = 5 for both WT and *qMCP*$^{-/-}$ mice. Source data are provided as a Source Data file.

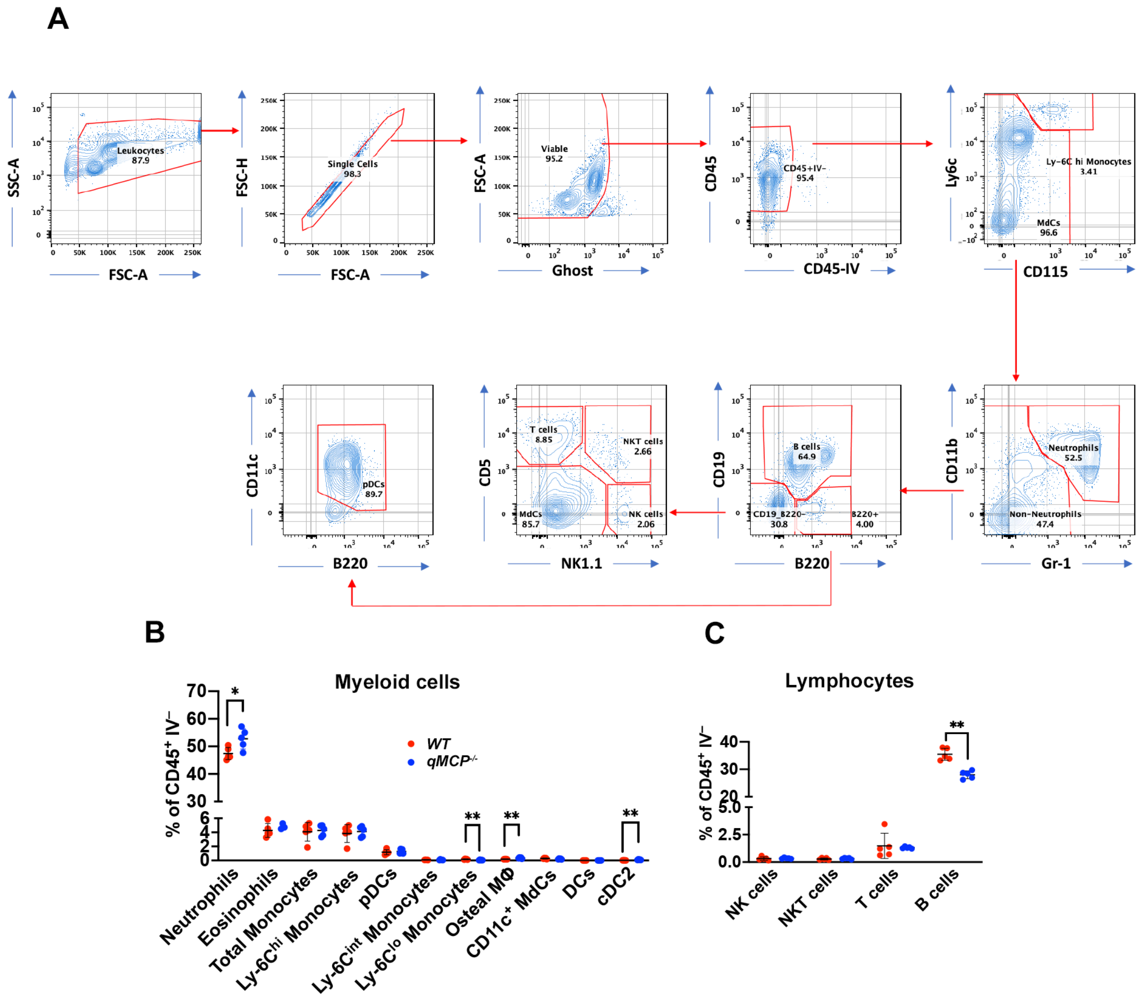

Supplementary Figure 5. Flow cytometric analysis of immune cells in the bone marrow of healthy adult mice. (A) Gating strategy identifying subsets of immune cells from of *WT* and *qMCP*$^{-/-}$ mice. (B) Quantification of myeloid cells. N = 5 for both *WT* and *qMCP*$^{-/-}$ mice. Data are presented as mean +/- SD. $P = 0.0242$, 0.0040, 0.0018, and 0.0032, respectively where asterisk are present. (C) Quantification of lymphoid cells. N = 5 for both WT and *qMCP*$^{-/-}$ mice. Data are presented as mean +/- SD. Two-sided Student's *t*-test for both B and C. $P = 0.0002$. * $P < 0.05$, ** $P < 0.01$. Source data are provided as a Source Data file.

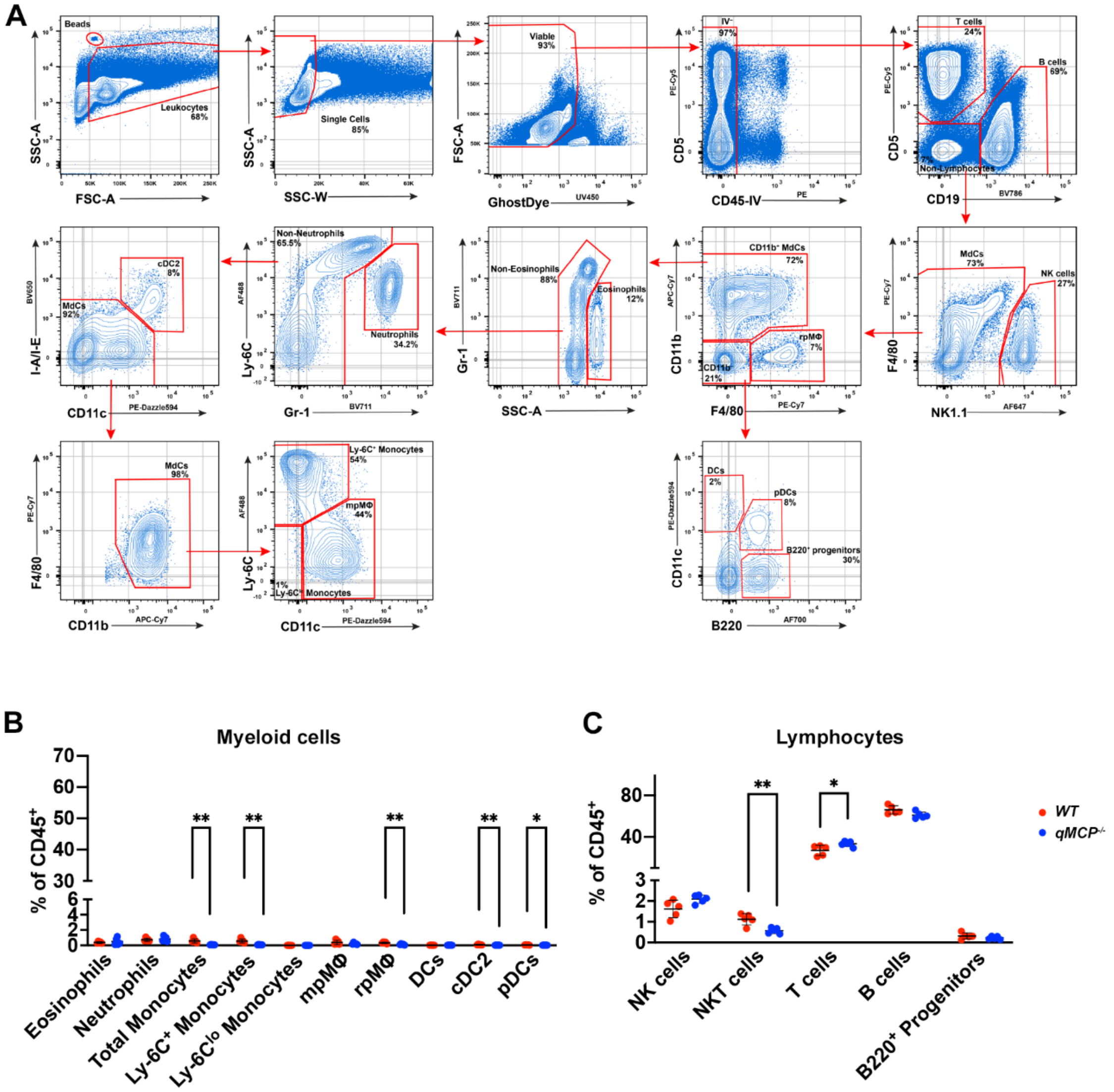

Supplementary Figure 6. Flow cytometric analysis of immune cells in spleen of healthy adult mice. (A) Gating strategy identifying subsets of immune cells of *WT* and *qMCP*$^{-/-}$ mice. (B) Quantification of myeloid cells. N = 5 for both *WT* and *qMCP*$^{-/-}$ mice. Data are presented as mean +/- SD. P = 0.0037, 0.0036, 0.0041, 0.0262, 0.0299, respectively. (C) Quantification of lymphoid cells. N=5 for both WT and *qMCP*$^{-/-}$ mice. Data are presented as mean +/- SD. Two-sided Student's *t*-test for both B and C. $P = 0.0042$, 0.0358, respectively. * $P < 0.05$, ** $P < 0.01$. Source data are provided as a Source Data file.

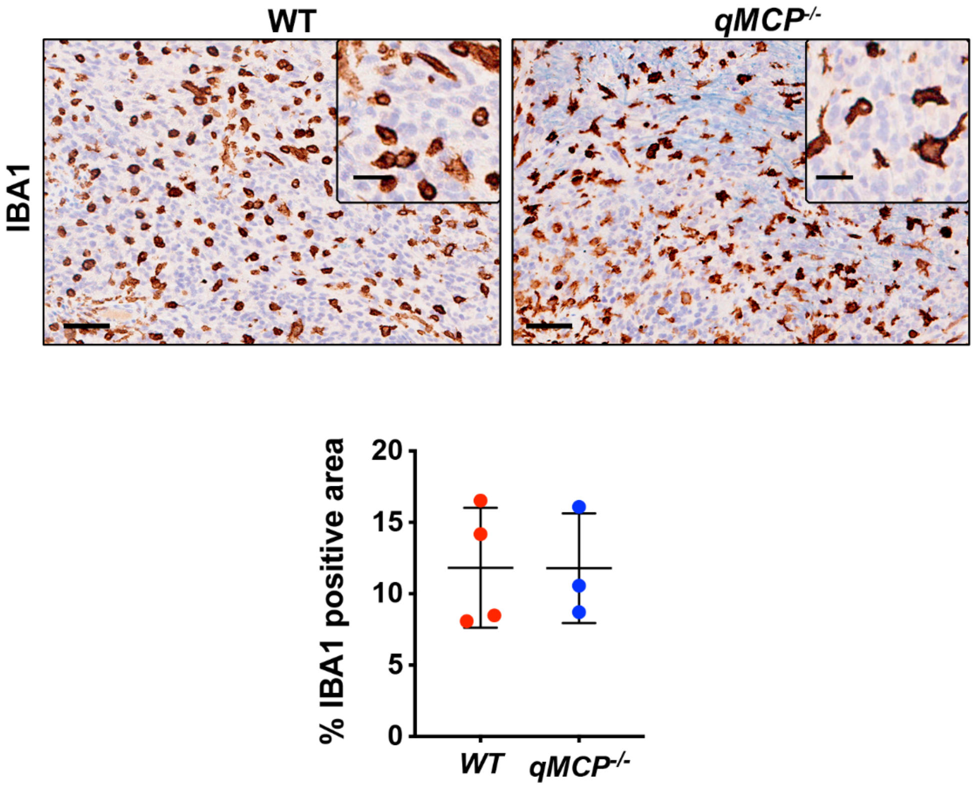

Supplementary Figure 7. Loss of MCPs from TME has no impact on tumor-associated macrophage positive area. Representative images and quantification immunohistochemistry for IBA1 in orthotopic transplant *PDGFB*-driven tumors generated in *WT* and *qMCP*$^{-/-}$ mice**.** Scale bar = 50 µm, scale bar in inset = 20 µm. Two-sided Student's *t*-test was applied. N = 4 and 3 for *WT* and *qMCP*$^{-/-}$ mice. Data are presented as mean +/- SD. Source data are provided as a Source Data file.

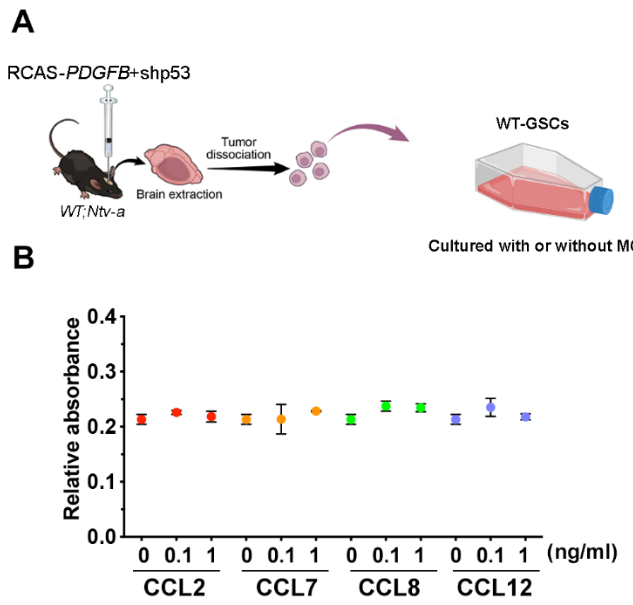

Supplementary Figure 8. Exogenous MCPs has no effect on the growth of *PDGFB*-driven GBM cells. (A) Schematic illustration of experimental paradigm of generating primary cultures from *PDGFB*-driven *de novo* tumors. (B) Quantification of cell growth by MTS assay in response to increasing doses of recombinant CCL2, CCL7, CCL8 and CCL12 stimulation. N = 3 independent experiments. Data are presented as mean +/- SD. Source data are provided as a Source Data file.

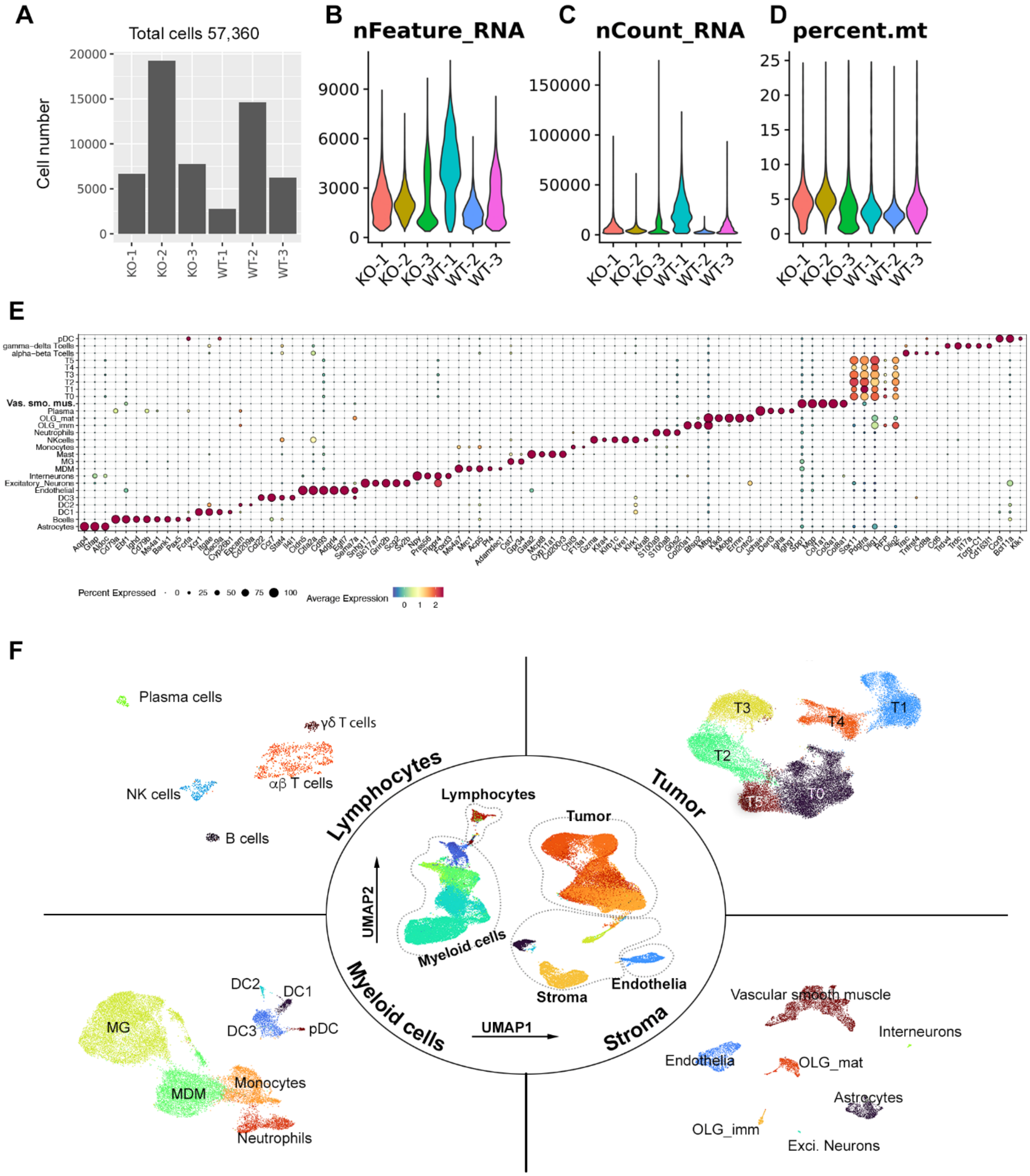

Supplementary Figure 9. Single-cell RNA seq analysis of tumors generated in *WT; Ntv-a* and *qMCP*$^{-/-}$*; Ntv-a* mice. (A) Total number of cells per samples after removing doublets. (B) Distribution of the number of genes (features) detected in each cell per sample. (C) Distribution of number of unique molecular identifiers (UMIs) in each cell per sample. (D) Distribution of percentage of mitochondrial genes expressed in each cell per sample. (E) Expression of select marker genes (x axis) for each annotated cell subset (y axis) colored by normalized expression level, where the size of dots represents the percentage of cells expressed. (F) UMAP

dimensionality reductions of the stratified cells. OLG: oligodendrocytes, imm: immature, mat: mature, Exci: excitatory. Source data are provided as a Source Data file.

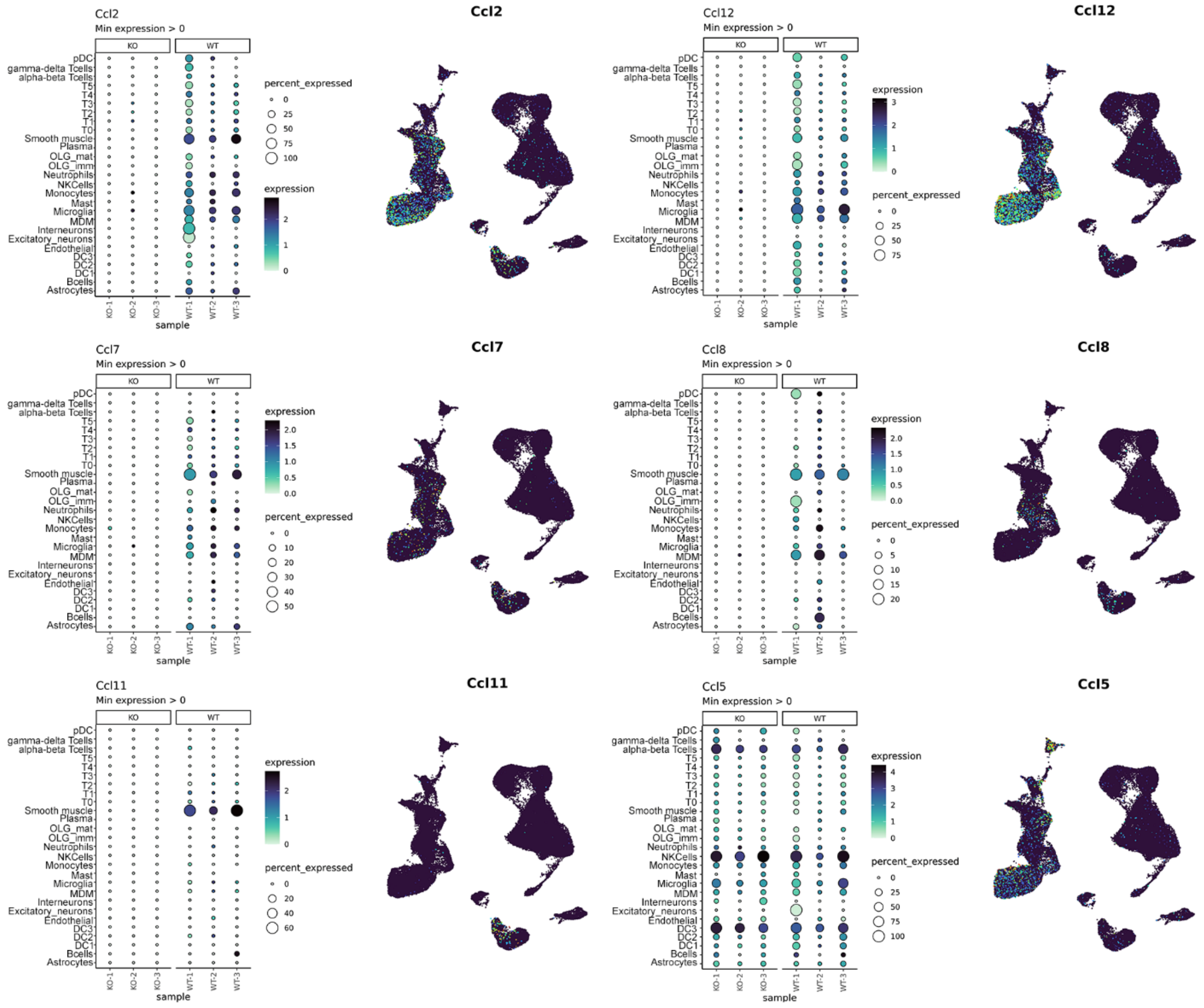

Supplementary Figure 10. Expression of MCP-related genes across cell types and samples. CCL expressions examined by scRNA-seq from tumors generated in *WT; Ntv-a* and *qMCP*$^{-/-}$*; Ntv-a* mice. Left in each panel: dot plots showing percentage of expressed cells (dot size) and normalized expression level (color gradient) across cell types (y-axes) and mouse tumor samples (x-axes). Right in each panel: UMAPs showing the expression of MCPs across all cells using the same UMAP embedding as in Figure 3A. Source data are provided as a Source Data file.

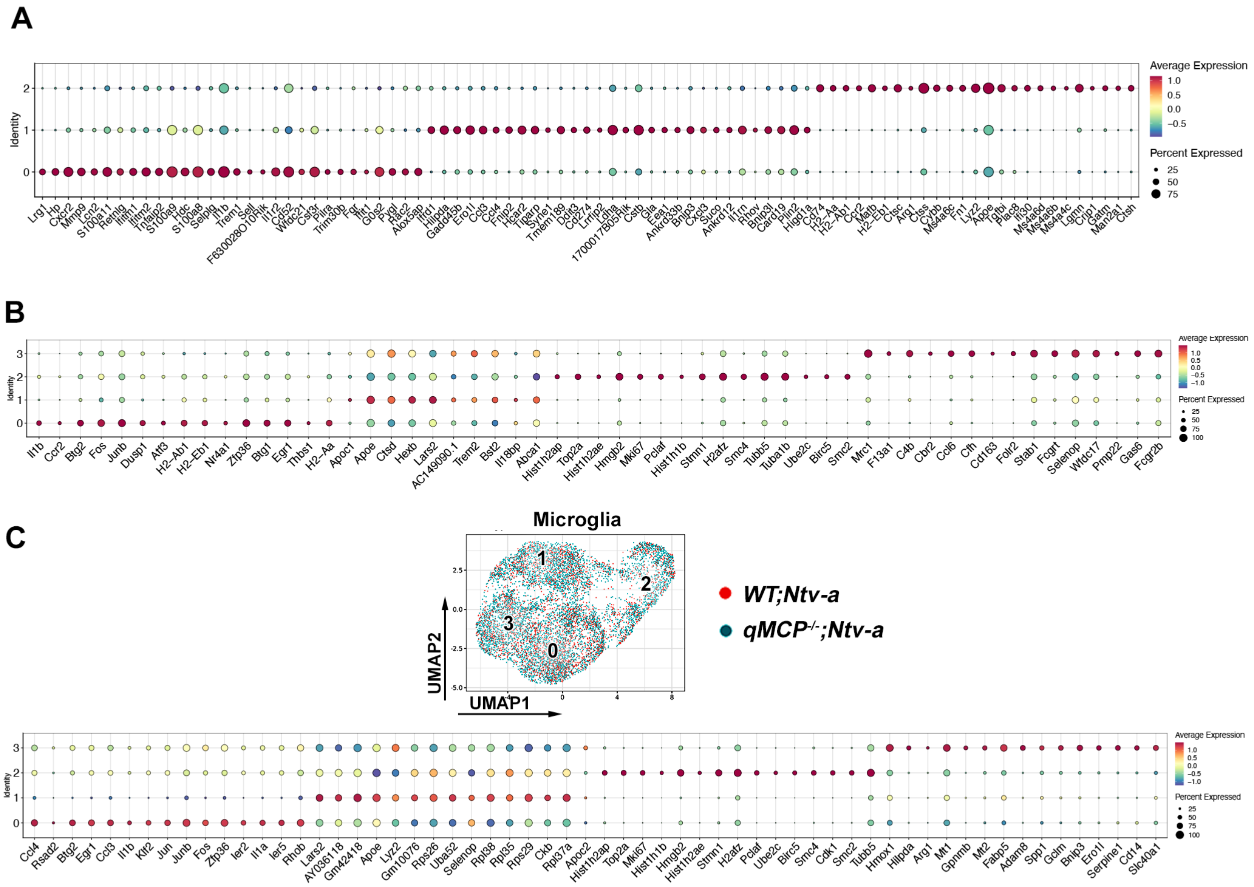

Supplementary Figure 11. UMAP dimensional reduction and subclass annotation of neutrophil, MDM and Mg in *WT; Ntv-a* and *qMCP$^{-/-}$; Ntv-a* mice examined by scRNA-seq. (A) Dot plot showing genes used to annotate neutrophil clusters. (B) Dot plot showing genes used to annotate MDM clusters. (C) UMAP and dot plots showing genes used to annotate Mg clusters. Source data are provided as a Source Data file.

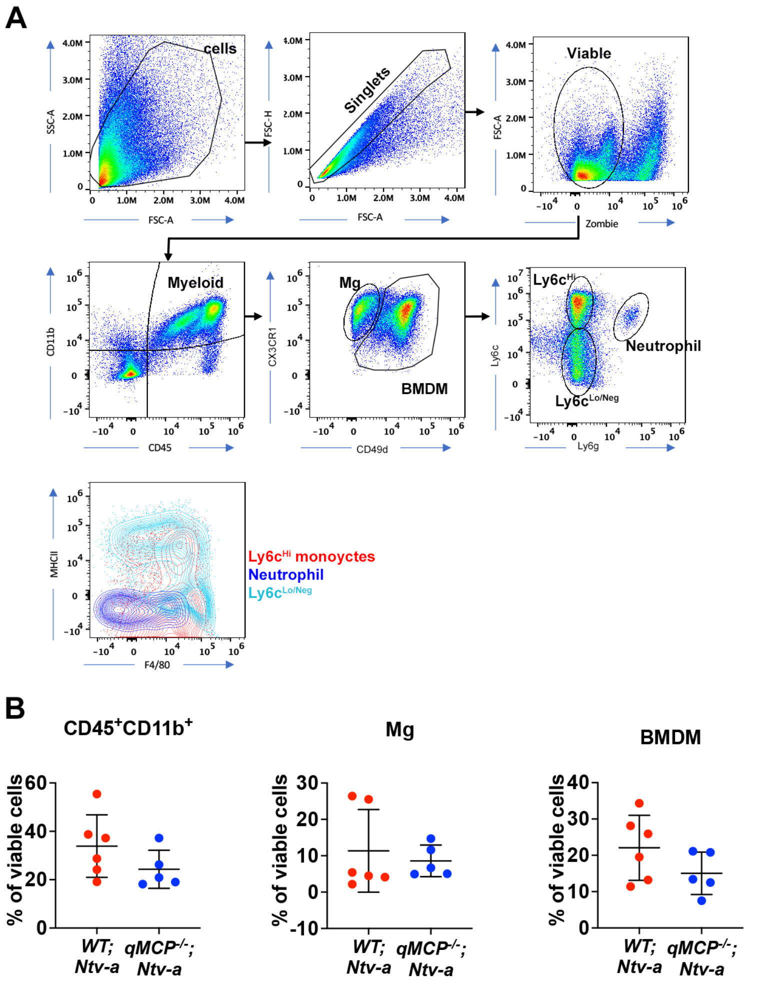

Supplementary Figure 12. Deletion of all MCPs has no impact on myeloid recruitment. (A) Gating strategy identifying myeloid cells by spectral flow cytometry. (B) Dot plots showing subsets of myeloid cells in the *PDGFB*-driven tumors generated in *WT; Ntv-a* and *qMCP*$^{-/-}$*; Ntv-a* mice. N = 6 and 5 for *WT* and *qMCP*$^{-/-}$ mice. Source data are provided as a Source Data file. Data are presented as mean +/- SD.

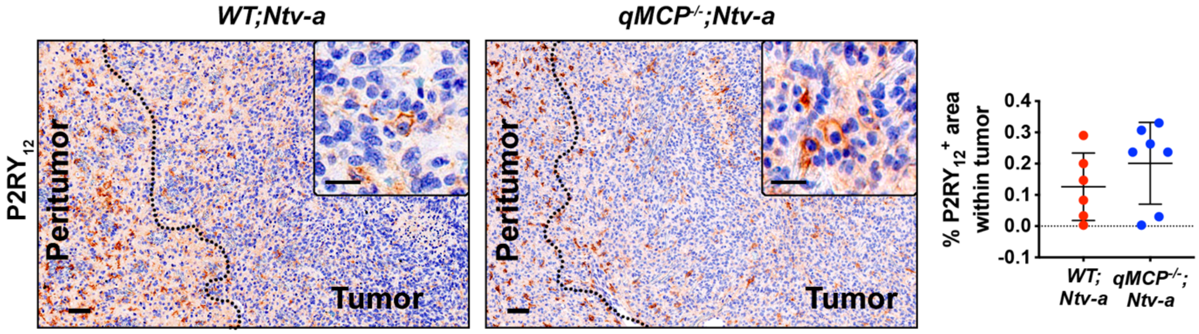

Supplementary Figure 13. Deletion of all MCPs has not impact on $P2RY_{12}$-positive area. Representative images and quantification of $P2RY_{12}$ staining for Mg by immunohistochemistry within *PDGFB*-driven tumors generated in *WT; Ntv-a* and *qMCP*$^{-/-}$*; Ntv-a* mice. N = 6 (*WT; Ntv-a*) and 7 (*qMCP*$^{-/-}$*; Ntv-a* mice). Data are presented as mean +/- SD. Source data are provided as a Source Data file.

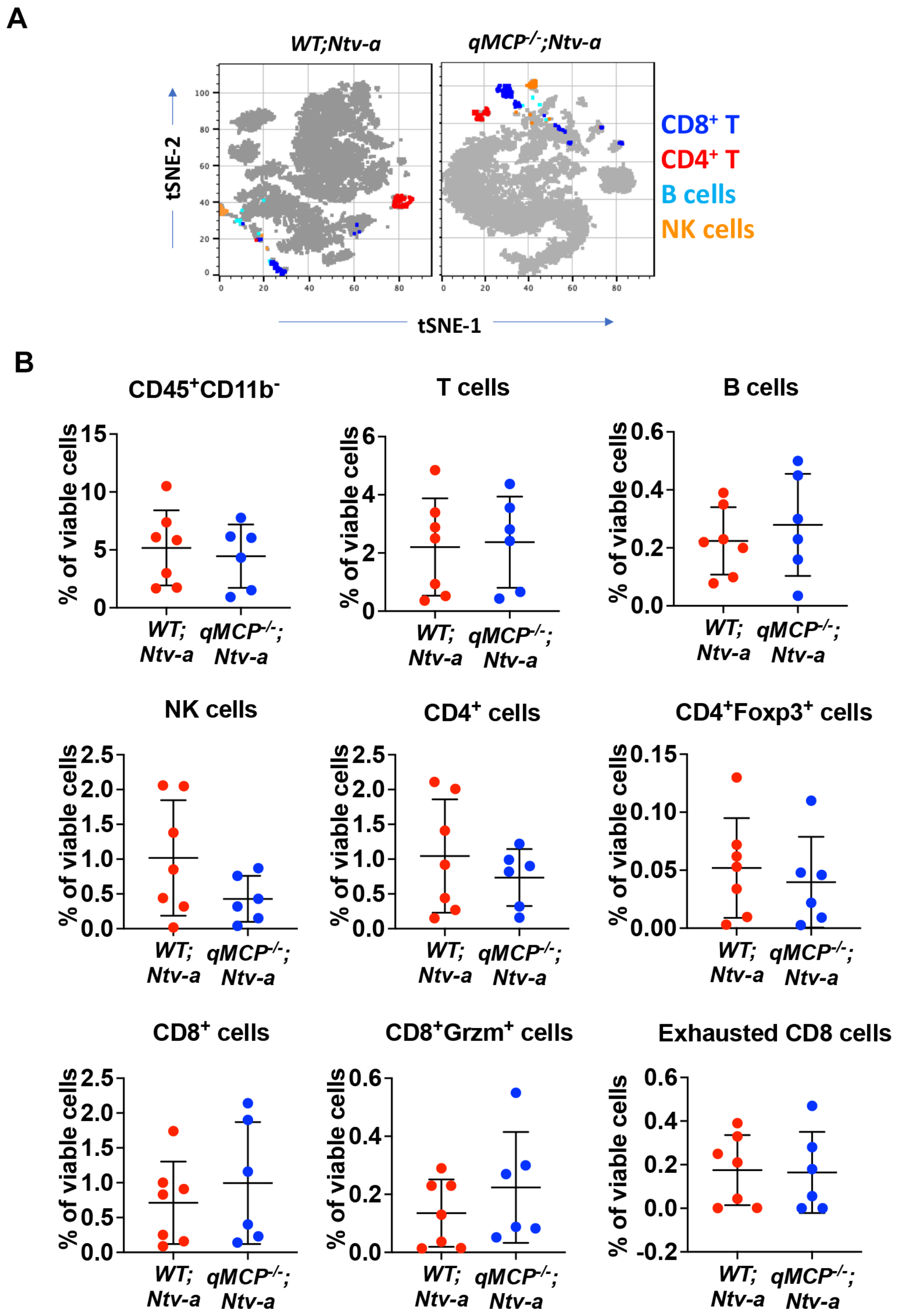

Supplementary Figure 14. Loss of MCPs has no effect on lymphoid populations in *PDGFB*-driven GBM. (A) tSNE plots showing lymphoid cells by spectral flow cytometry in *PDGFB*-driven GBM generated in *WT;Ntv-a* and *qMCP*$^{-/-}$*; Ntv-a* mice. (B) Dot plots quantifying subsets of lymphoid cells in the tumors. N = 7 (*WT; Ntv-a*) and 6 (*qMCP*$^{-/-}$*; Ntv-a* mice). Data are presented as mean +/- SD. Source data are provided as a Source Data file.

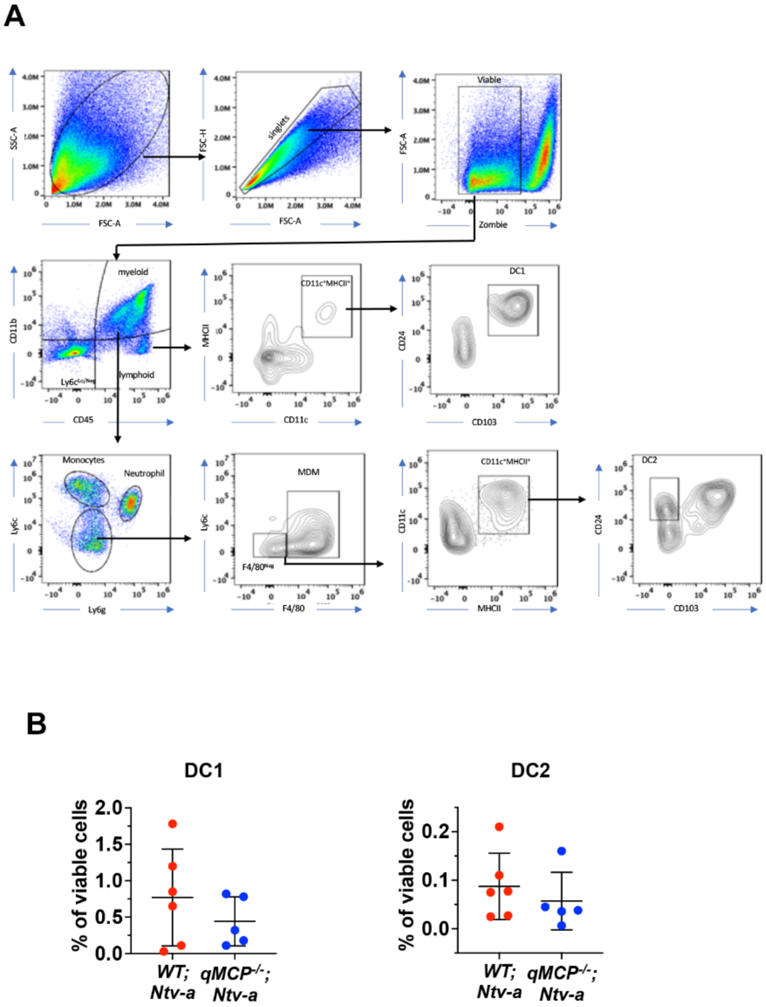

Supplementary Figure 15. Loss of MCPs has no impact on number of dendritic cells (DC) in GBM. (A) Gating strategy identifying DCs by spectral flow cytometry in *PDGFB*-driven GBM generated in *WT;Ntv-a* and *qMCP*$^{-/-}$*;Ntv-a* mice. (B) Dot plots quantifying DCs in the tumors. N = 6 (*WT; Ntv-a*) and 5 (*qMCP*$^{-/-}$*; Ntv-a* mice). Data are presented as mean +/- SD. Source data are provided as a Source Data file.

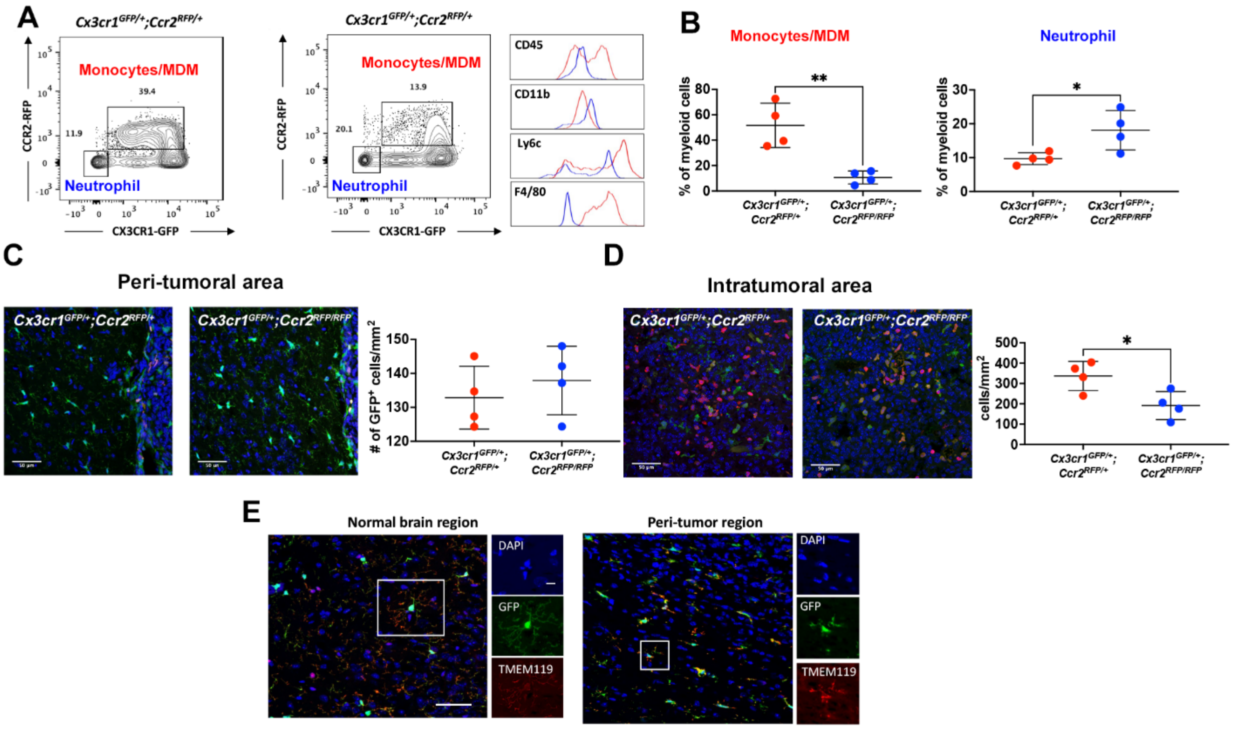

Supplementary Figure 16. Loss of *Ccr2* receptor results in reduced presence of monocytes/MDM and increase in neutrophils with no changes in peritumoral Mg. (A) Representative images of gating myeloid cells in *PDGFB*-driven orthotopic transplant tumors generated in *Cx3Cr1*$^{GFP/WT}$*; Ccr2*$^{RFP/WT}$ *and Cx3Cr1*$^{GFP/WT}$*; Ccr2*$^{RFP/RFP}$ adult mice. (B) Quantification dot plots of monocyte/MDM and neutrophils. N=4 independent mice for both genotypes. Data are presented as mean +/- SD. P = 0.0040 and 0.0324, respectively. (C) Representative images for immunofluorescent staining for GFP (CX3CR1), RFP (CCR2) and DAPI (nuclei) and the corresponding quantifications of GFP^{+} cells in peri-tumoral areas. N = 4 independent mice for both genotypes. Data are presented as mean +/- SD. (D) Representative images for immunofluorescent staining for GFP (CX3CR1), RFP (CCR2) and DAPI (nuclei) and the corresponding quantifications of GFP^{+}RFP^{+} cells within the tumor. N = 4 independent mouse for both genotypes. Data are presented as mean +/- SD. P = 0.0260. (E) Representative images of GFP and TREM119 staining for Mg in contralateral hemisphere and peritumoral region. Scale bar = 50 μm. Scale bar of inset = 10 μm. Two-sided Student's *t*-test for both B and D. * $P < 0.05$, ** $P < 0.01$. Source data are provided as a Source Data file.

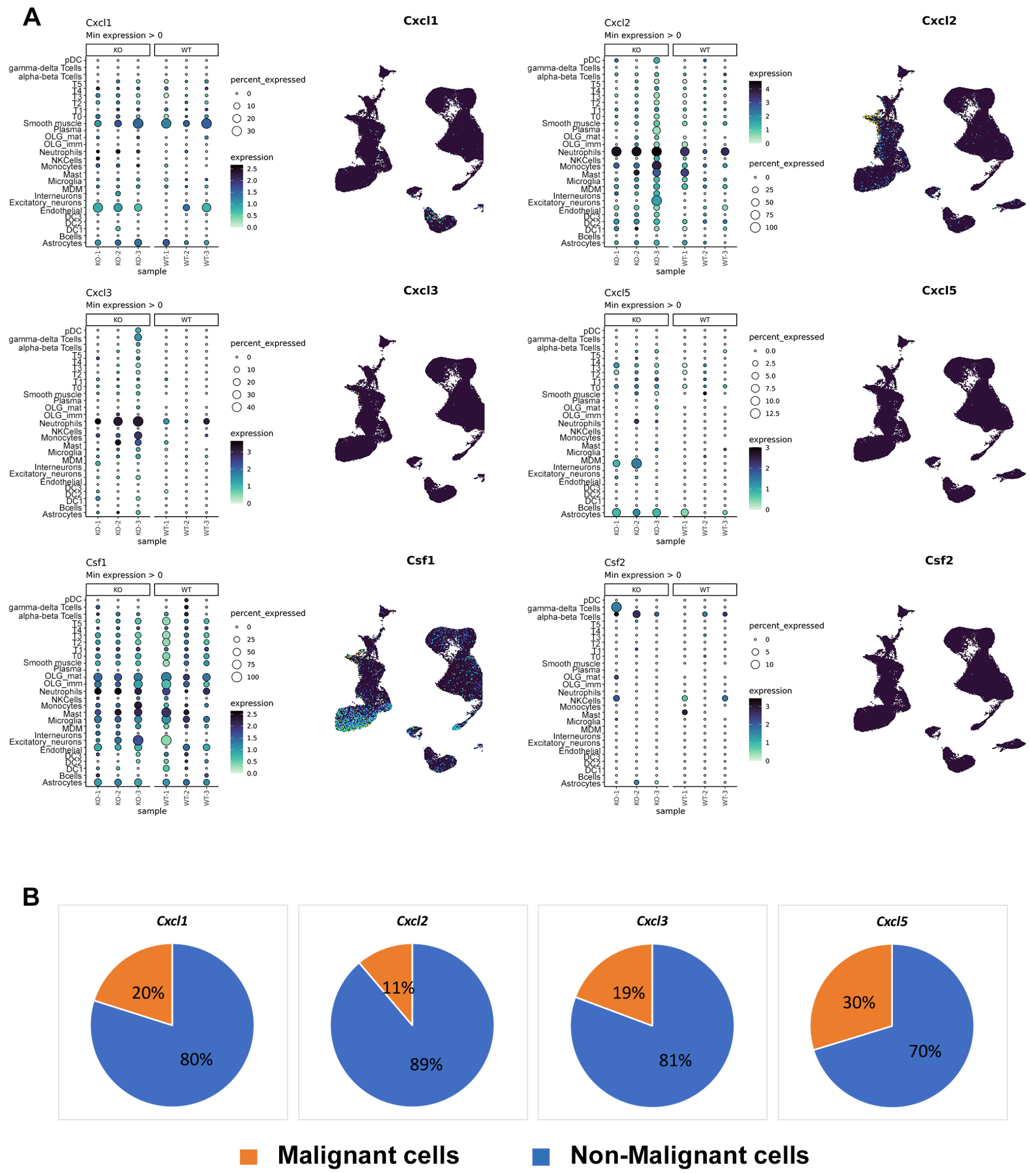

Supplementary Figure 17. *Cxcl2* and *Cxcl3* expression is increased in neutrophils in monocyte abolished *PDGFB*-driven tumors. (A) *Cxcl* expressions across cell types and genotypes examined by scRNA-seq. Left in each panel: dot plots showing percentage of expressed cells (dot size) and normalized expression level (color gradient) across cell types (y-axes) and mouse tumor samples (x-axes). Right in each panel: UMAPs showing the expression of *Cxcls, Csf1* and *Csf2* across all cells using the same UMAP embedding as in Figure 2B. (B) Pie chart quantification of *Cxcl* expression between non-malignant and malignant cells based on data shown in panel A. Source data are provided as a Source Data file.

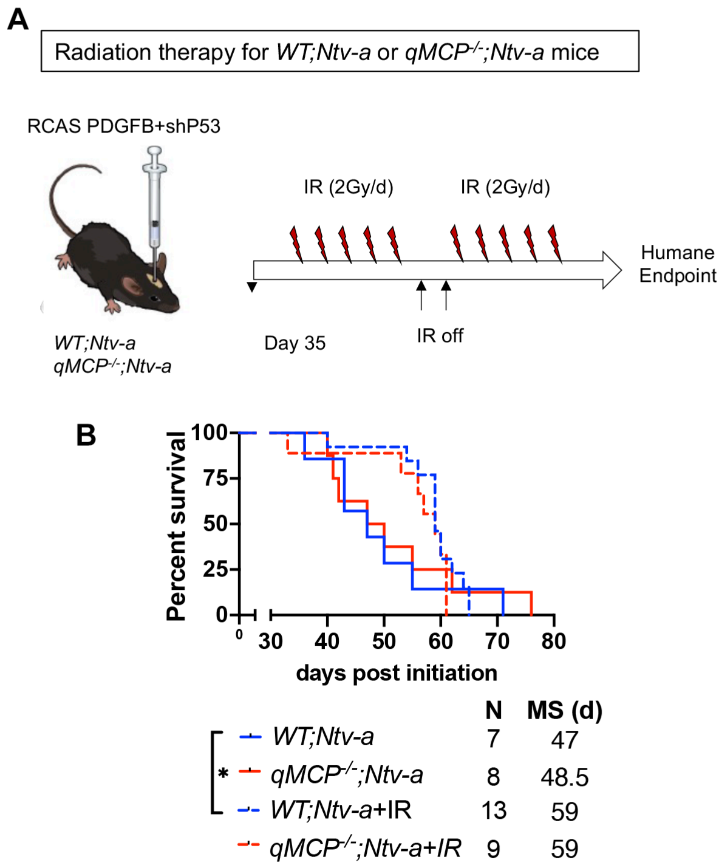

Supplementary Figure 18. RT prolongs the survival of PDGFB-driven tumors in *WT;Ntv-a* but *qMCP$^{-/-}$;Ntv-a* mice. (A) Illustration of experimental design. IR = irradiation. (B) Kaplan-Meier survival curves of tumor-bearing *WT; Ntv-a* and *qMCP$^{-/-}$; Ntv-a* mice with or without RT. Gehan-Berslow-Wilcoxon (GBW) test. $P = 0.0169$ between *WT; Ntv-a* and *WT; Ntv-a*+IR (* $P < 0.05$) by GBW test. MS = median survival, N = number of mice. RT = radiation therapy. IR = Irradiation. Source data are provided as a Source Data file.

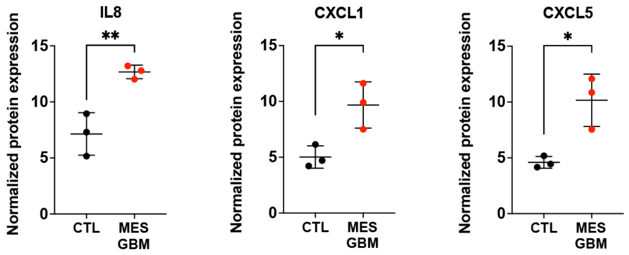

Supplementary Figure 19. Neutrophil recruitment chemokine levels are increased in MES human GBM. Normalized protein expressions of IL8, CXCL1 and CXCL5 examined by Olink proteomic assay between normal brain as a control and MES hGBM samples. Two-sided Student's *t*-test. $P = 0.0085$, 0.0247, and 0.0160, respectively. * $P < 0.05$, ** $P < 0.01$. N = 3 independent tissue samples for both control and MES GBM samples. Data are presented as mean +/- SD. Source data are provided as a Source Data file.

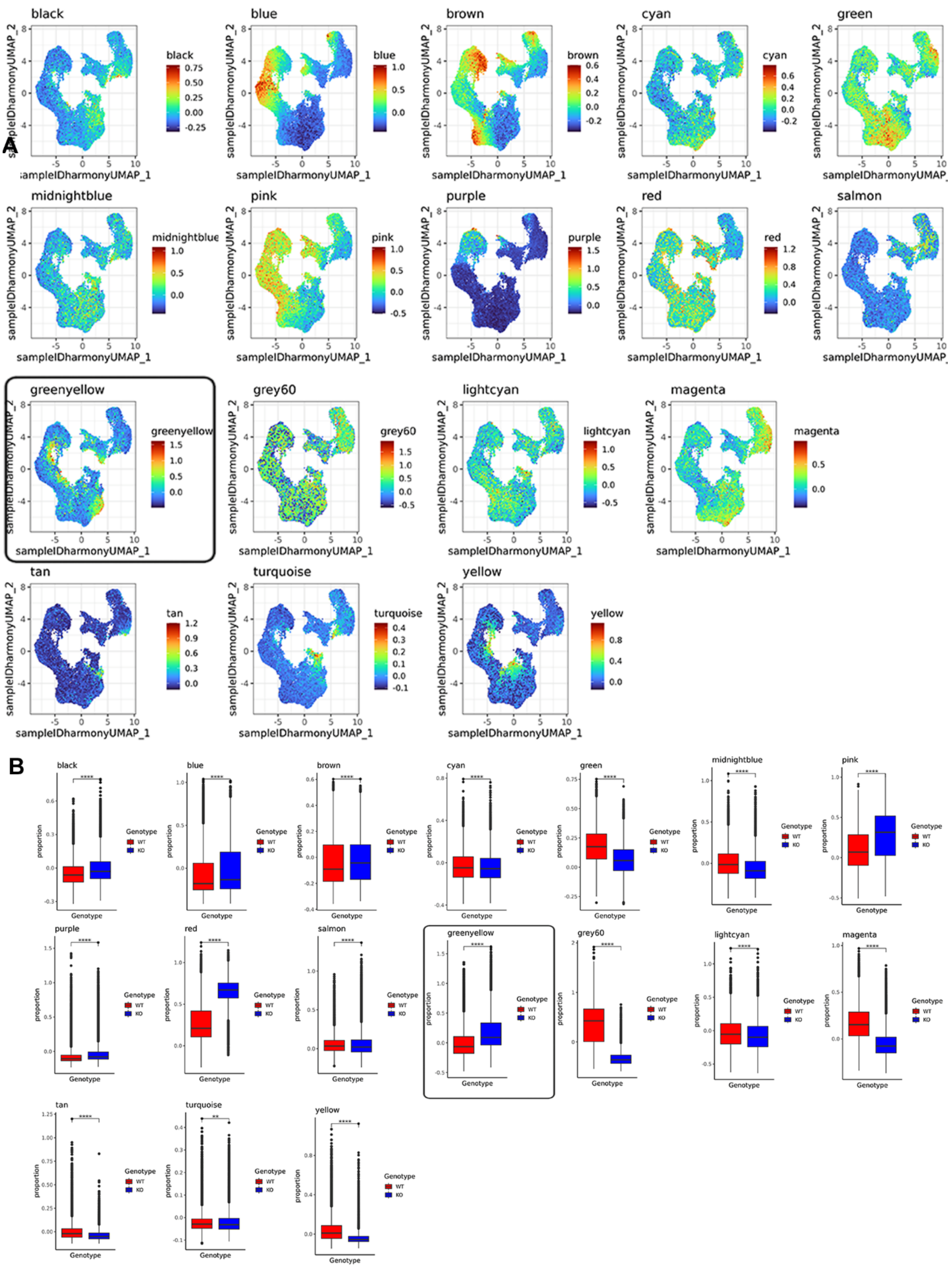
A
black
blue
brown
cyan
green
midnightblue
pink
purple
red
salmon
greenyellow
grey60
lightcyan
magenta
tan
turquoise
yellow
sampleIDharmonyUMAP_1
sampleIDharmonyUMAP_2
B
Genotype
WT
KO
proportion
****
**

Supplementary Figure 20. WGCNA of scRNA-seq data identified "Greenyellow" cancer co-expression module increase in qMCP-deleted tumors. (A) UMAPs of cancer gene co-expression module scores for each cancer module identified by WGCNA. (B) Box plots of average module scores per sample split by genotypes (red vs. blue) across all cancer cells. "Greenyellow" module (outlined) show pronounced difference between PDGFB mGBM generated in *WT; Ntv-a* (red) and *qMCP*$^{-/-}$*; Ntv-a* (blue) mice. Two-sided Student's *t*-test. **** $P < 0.0001$. Source data are provided as a Source Data file.

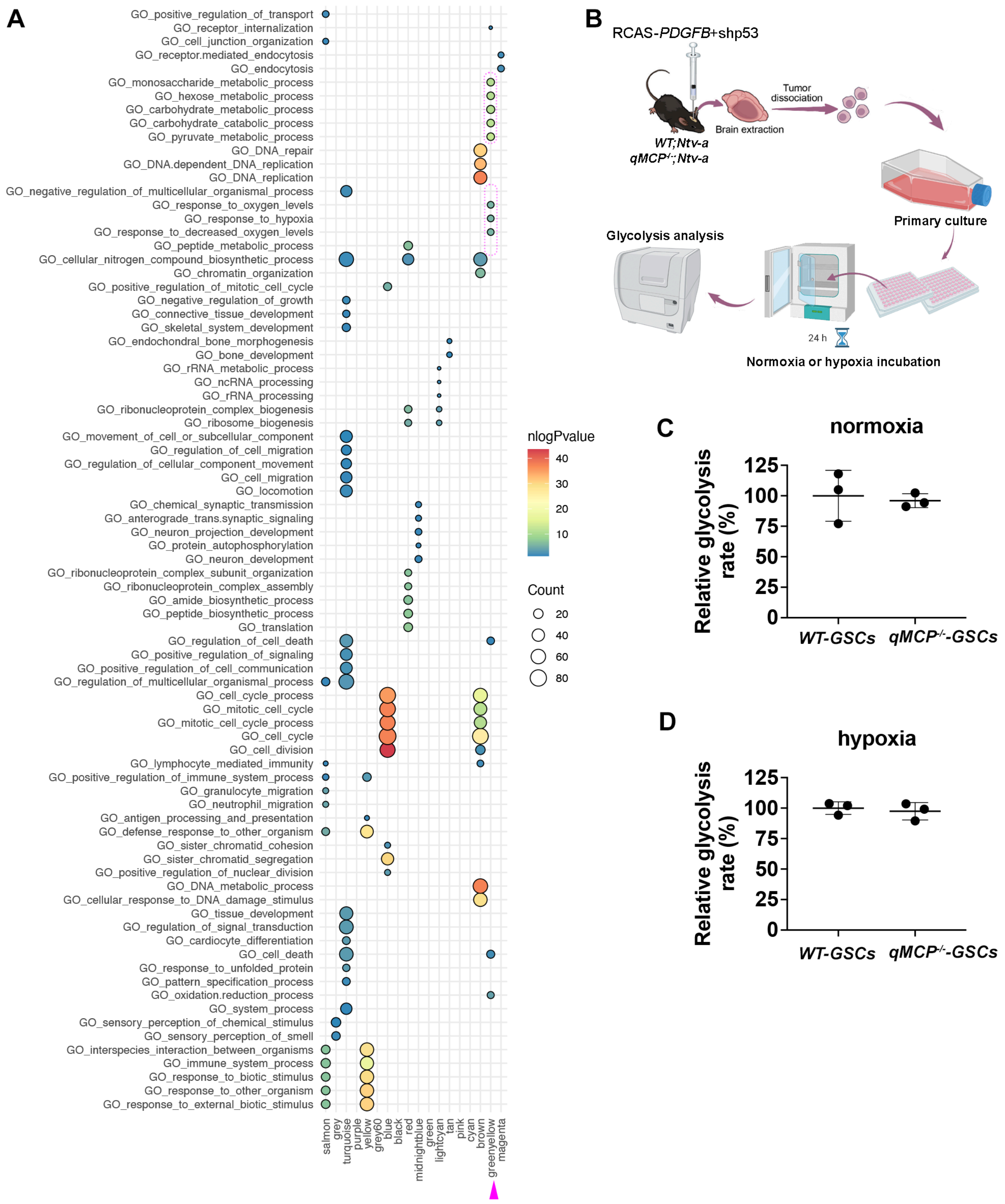

Supplementary Figure 21. Top enriched gene ontology (GO) terms for co-expressed genes identified in each WGCNA module. (A) Dot plot showing the top five GO biological process terms enriched for genes in each WGCNA module. "Greenyellow" module genes were enriched for hypoxic response and glycolysis GO terms (dotted lines). Size of dots represents the number of genes in the enrichment and color of dots present the -log(P-value) of the pathway enrichment. Benjamini-Hochberg adjusted (BH-adjusted) hypergeometric test was used. (B) Schematic illustration of experimental paradigm for C and D. (C) Relative glycolysis rate in primary tumor

cell cultures at 24 hrs. under normoxia (18% O_2) condition. N = 3 independent primary cell cultures for both genotypes. Data are presented as mean +/- SD. (D) Relative glycolysis rate in primary cultures at 24 hrs. under hypoxia condition (2% O_2). N = 3 independent primary cell cultures for both genotypes. Data are presented as mean +/- SD. Source data are provided as a Source Data file.

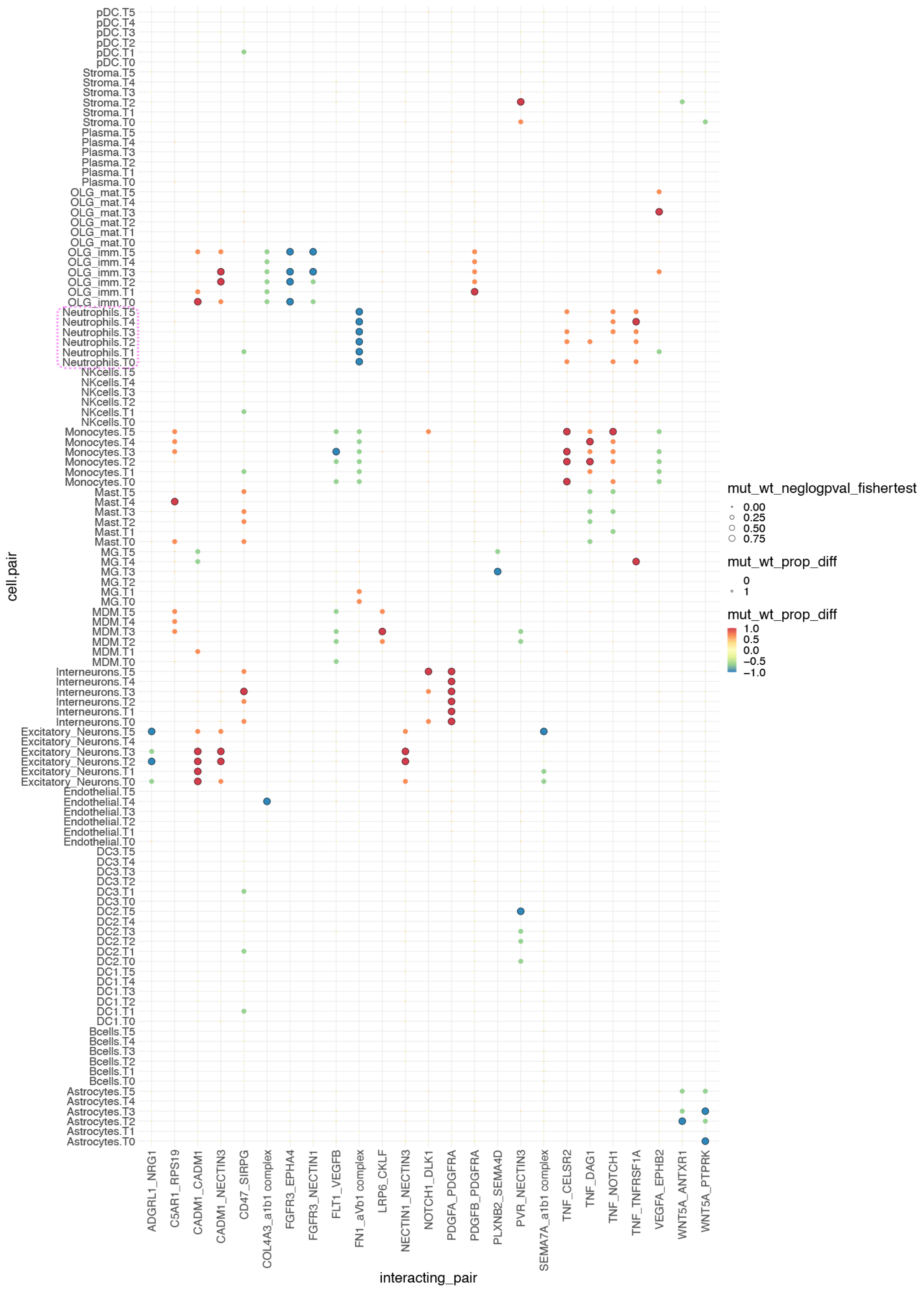

Supplementary Figure 22. Differential ligand-receptor analysis between KO and WT samples. Dot plot showing the differential enrichment of interactions between corresponding cell-cell (y-axis) and ligand-receptor pairs (x-axis). Interactions were identified by CellPhoneDB. Color of dots represents the proportional difference in enrichment between *WT; Ntv-a* (blue) and *qMCP*$^{-/-}$*; Ntv-a* (red) samples, size of dot represents the -log (*P*-value) of the differential enrichment (Fisher exact test), and dark circle outline represents a significant differential interaction (*p < 0.05). Source data are provided as a Source Data file.

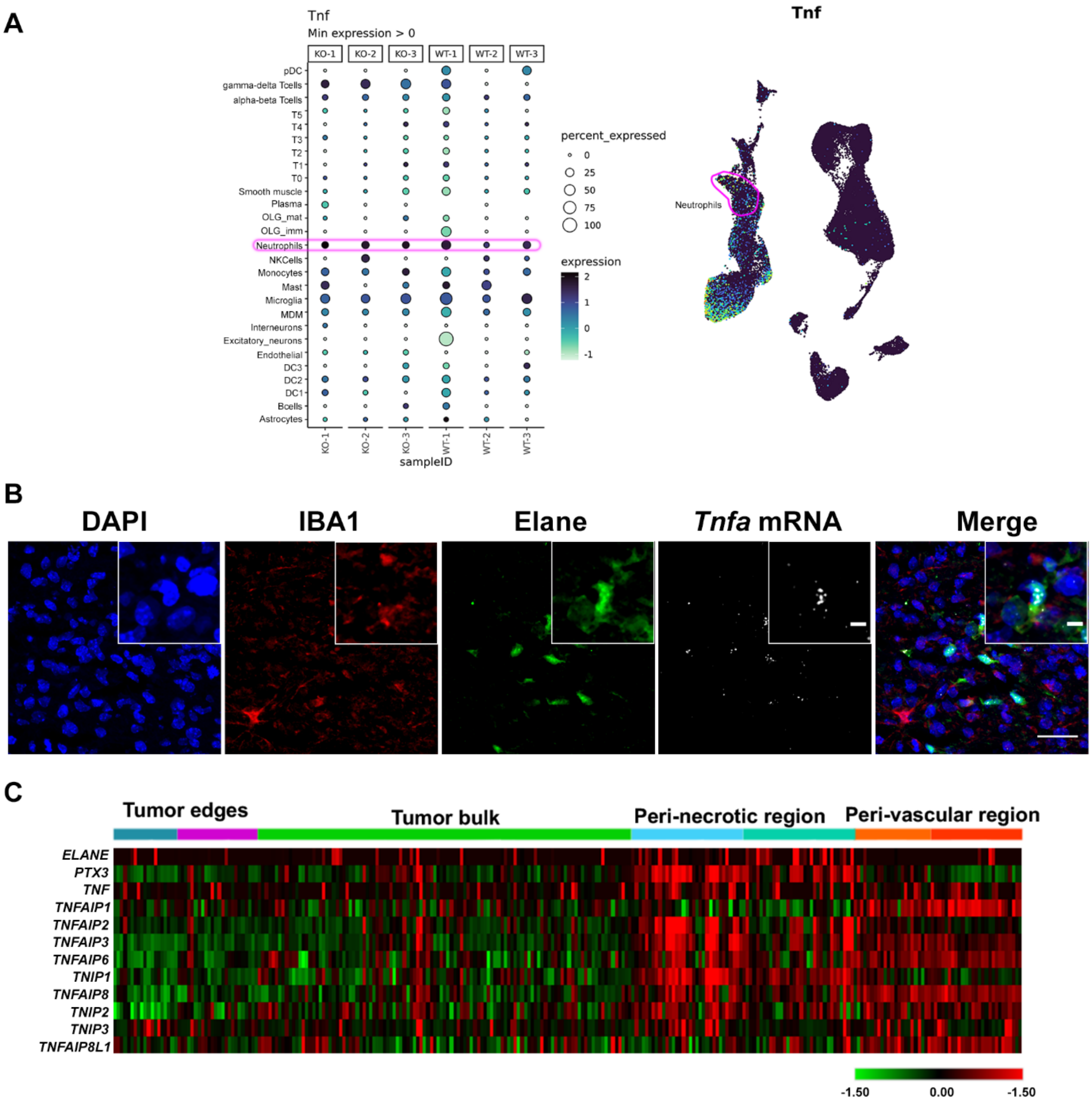

Supplementary Figure 23. Neutrophils are major source of *Tnfa* in *PDGFB*-driven GBM. (A) *Tnfa* expression across cell types and genotypes examined by scRNA-seq. Left: dot plots showing percentage of expressed cells (dot size) and normalized expression level (color gradient) across cell types (y-axes) and mouse tumor samples (x-axes). Right: Expression of *Tnfa* across all cells visualized by UMAP dimensionality reduction. The highest % expression is shown in neutrophils (circled in pink). Contribution of neutrophils to *Tnfa* expression highlighted in pink. (B) Representative images of FISH with *Tnfa* RNA-probe (gray) combined with IF staining for IBA1 (red) for macrophages and Elane (green) for neutrophils and DAPI (blue) for nuclei. Merged images show that RNA expression for *Tnfa* co-localizes with Elane^{+} neutrophils. Scale bar = 25 µm. scale bar of inset = 5 µm. Images represent three independent experiments. (C) IVYGap analysis of spatial expression of Elane and TNF family members. Source data are provided as a Source Data file.

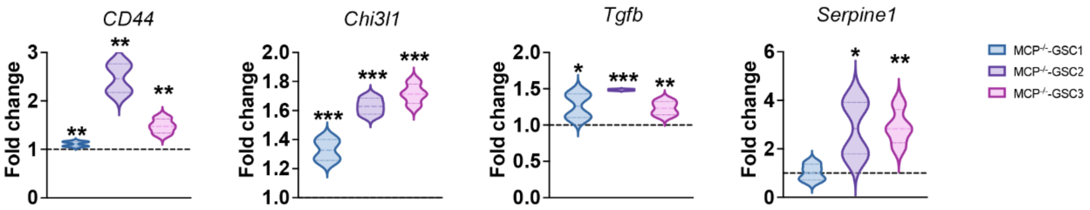

Supplementary Figure 24. TNF-α stimulation of qMCP-deficient GSC cultures induces expression of MES signature genes. Quantitative real-time PCR for expression of MES signature genes in qMCP-deficient primary GSC cultures in *WT-GSCs* treated with 10 ng/mL of TNF-α for 48 hrs. Two-sided Student's paired *t*-test compared to non-treated cells. * $P < 0.05$, ** $P < 0.001$, *** $P < 0.0001$. Source data are provided as a Source Data file.

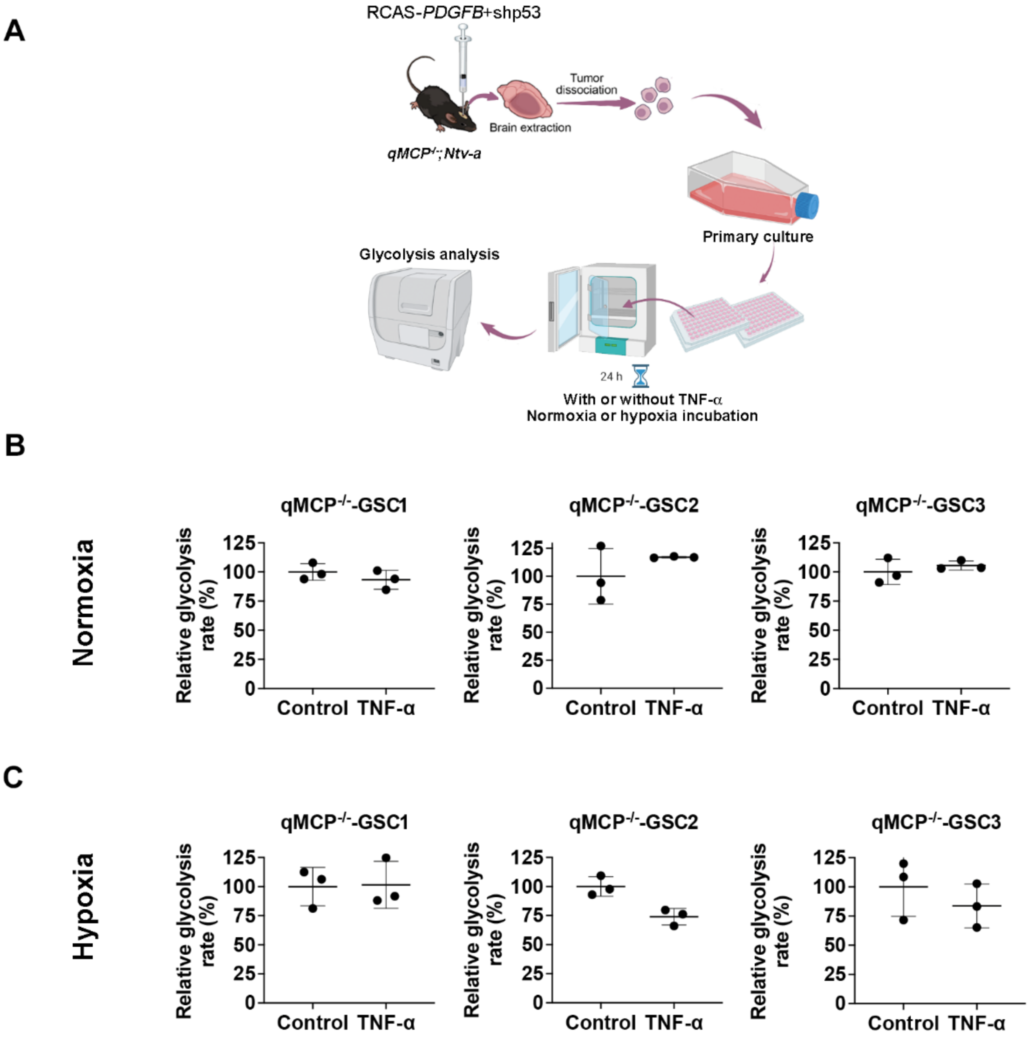

Supplementary Figure 25. **TNF-α stimulation of qMCP-deficient GSC cultures has no effect on glycolysis rate. (**A) Schematic illustration of experimental paradigm. (B) Relative glycolysis rate in primary qMCP-deficient GSC cultures at 24 hrs. under normoxia (18% O_2) condition with or without TNF-α (10 ng/ml). N = 3 independent primary cell cultures for both conditions. Data are presented as mean +/- SD. **(C)** Relative glycolysis rate in primary qMCP-deficient GSC cultures at 24hrs. under hypoxia (2% O_2) condition with or without TNF-α (10 ng/ml). Data are presented as mean +/- SD. N = 3 independent primary cell cultures for both conditions. Source data are provided as a Source Data file.

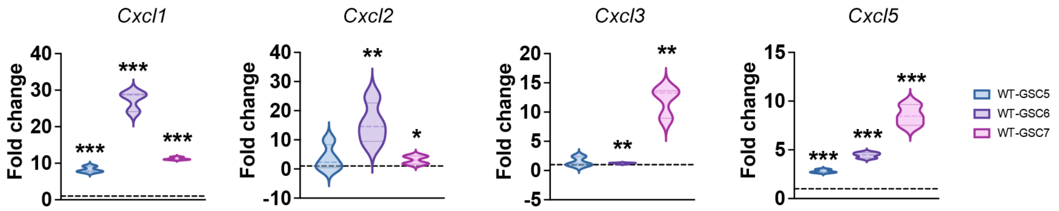

Supplementary Figure 26. TNF-α stimulation induces expression of neutrophil recruitment chemokines in *PDGFB*-driven GBM primary cultures. Quantitative real-time PCR for expression of neutrophil recruitment chemokines in primary *PDGFB*-driven WT GBM tumor cell cultures in response to TNF-α (10 ng/ml, 48 hrs.) compared to non-stimulated controls. Two-sided Student's *t*-test comparing to baselines (dotted lines on graphs). * $P < 0.05$, ** $P < 0.001$. Source data are provided as a Source Data file.

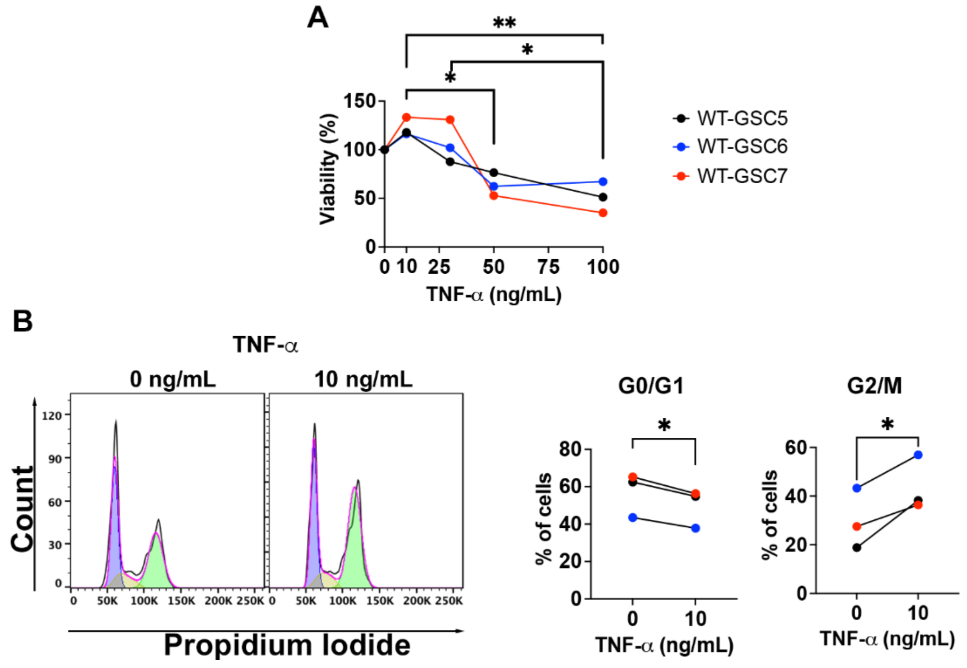

Supplementary Figure 27. TNF-α stimulation of *PDGFB*-driven *WT* GBM primary cultures at low doses increases cell growth and high does induce cell death. (A) MTS assay for TNF-α dose response at 48 hrs. post treatment in three primary WT-GSCs. P = 0.0104 (10 vs. 50 ng/ml), 0.0031 (10 vs. 100 ng/ml), and 0.0139 (25 vs. 100 ng/ml), (B) Representative images for gating for propidium iodide (PI) staining for cell cycle analysis of WT-GSCs treated with 0 and 10 ng/ml TNF-α for 48hrs. P = 0.0154 and 0.0442, respectively. Two-way ANOVA with Tuckey's post hoc test for A, and Two-sided Student's paired t-test for B. * $P < 0.05$, ** $P < 0.001$. Source data are provided as a Source Data file.

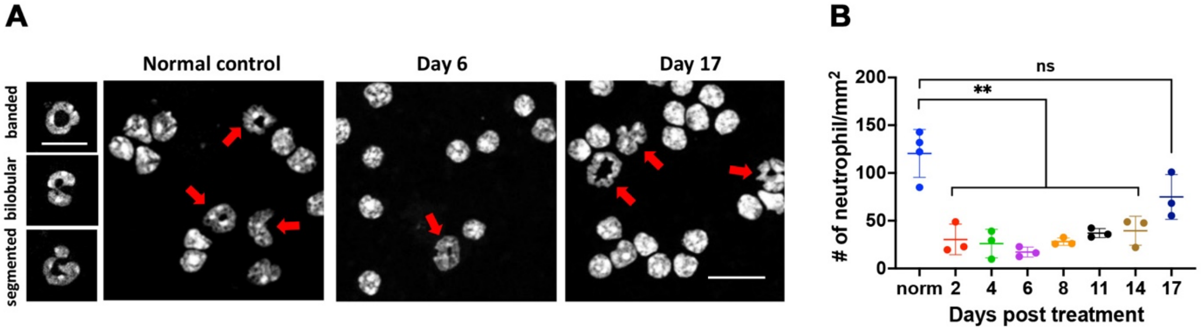

Supplementary Figure 28. Depletion of neutrophil by anti-Ly6g antibody is transient. (A) Morphology of neutrophils analyzed by Cytospin and fluorescent microscopy. Scale bar = 20 µm. Scale bar of inset = 10 µm. (B) quantification of blood neutrophils over time during anti-Ly6g antibody treatment in healthy adult mice. ** $P < 0.001$ by one-way ANOVA followed by Tukey's *post-hoc* test. Source data are provided as a Source Data file. N = 4 independent mouse for norm control and 3 for treated groups. Data are presented as mean +/- SD.

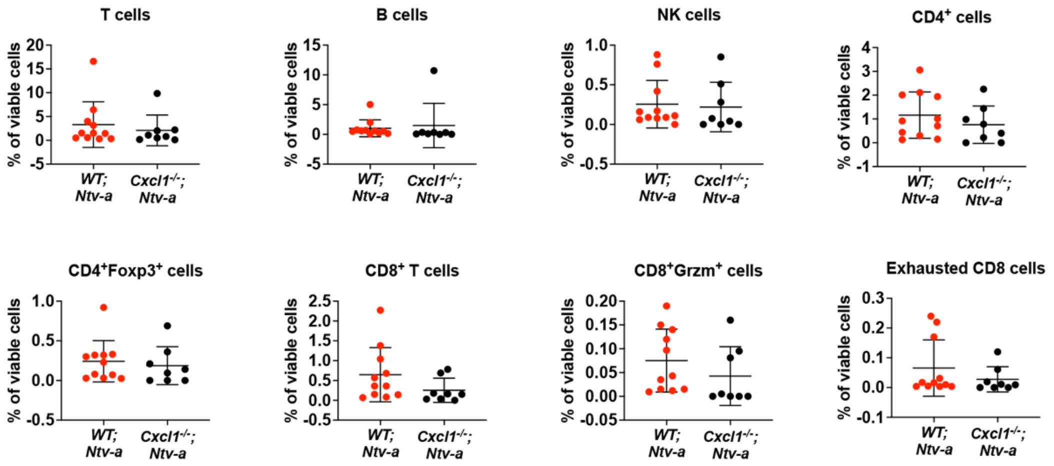

Supplementary Figure 29. *Cxcl1* loss has no effect on lymphoid populations in Nf1-silenced GBM. Quantification dot plots of lymphoid subsets of *Nf1*-silenced GBM generated in *WT; Ntv-a* and *Cxcl1*$^{-/-}$*; Ntv-a* mice by spectral flow cytometry. N = 11 and 8 independent mice for *WT* and *Cxcl1*$^{-/-}$ groups. Data are presented as mean +/- SD. Source data are provided as a Source Data file.

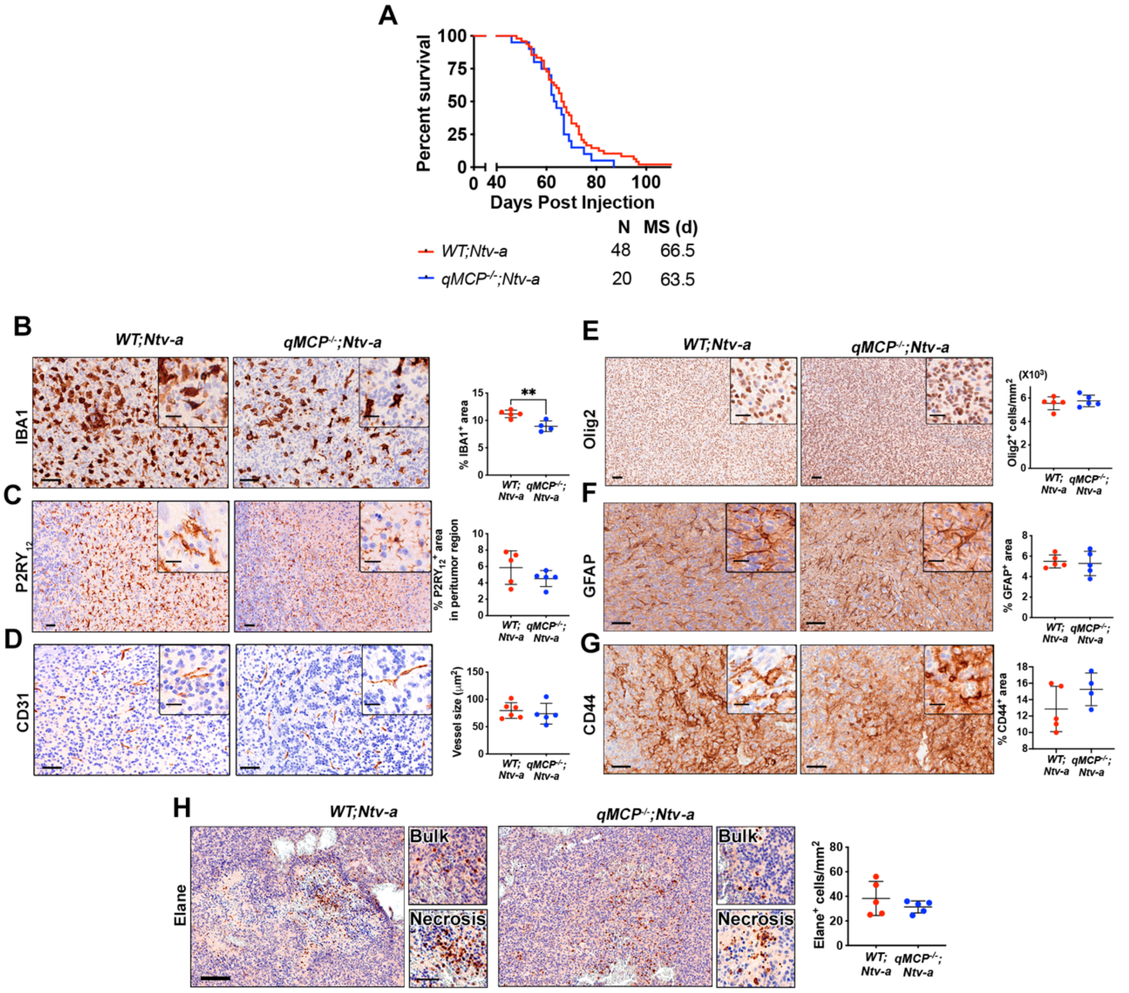

Supplementary Figure 30. Abolishing monocyte recruitment has no effect on neutrophil influx and survival of Nf1-silenced GBM-bearing mice. (A) Kaplan-Meier survival curves of *Nf1*-silenced tumors generated in *WT; Ntv-a* and *qMCP*$^{-/-}$*; Ntv-a* mice. (B-H) Representative images and quantification graphs of immunohistochemistry for IBA1 (B, *P* = 0.0047, N=5 and 4 for *WT; Ntv-a* and *qMCP*$^{-/-}$*; Ntv-a* mice), $P2RY_{12}$ (C, N = 5 for both *WT; Ntv-a* and *qMCP*$^{-/-}$*; Ntv-a* mice), CD31 (D, N = 6 and 5 for *WT; Ntv-a* and *qMCP*$^{-/-}$*; Ntv-a* mice), OLIG2 (E, N = 5 for both *WT; Ntv-a* and *qMCP*$^{-/-}$*; Ntv-a* mice), GFAP (F, N = 5 for both *WT; Ntv-a* and *qMCP*$^{-/-}$*; Ntv-a* mice), CD44 (G, N = 5 and 4 for *WT; Ntv-a* and *qMCP*$^{-/-}$*; Ntv-a* mice) and Elene (H, N = 5 for both *WT; Ntv-a* and *qMCP*$^{-/-}$*; Ntv-a* mice) in GBM sections generated from A**.** Data are presented as mean +/- SD. Two-sided Student's *t*-test. ** $P < 0.001$. Scale bar = 50 μm, scale bar in inset = 20 μm. Source data are provided as a Source Data file.

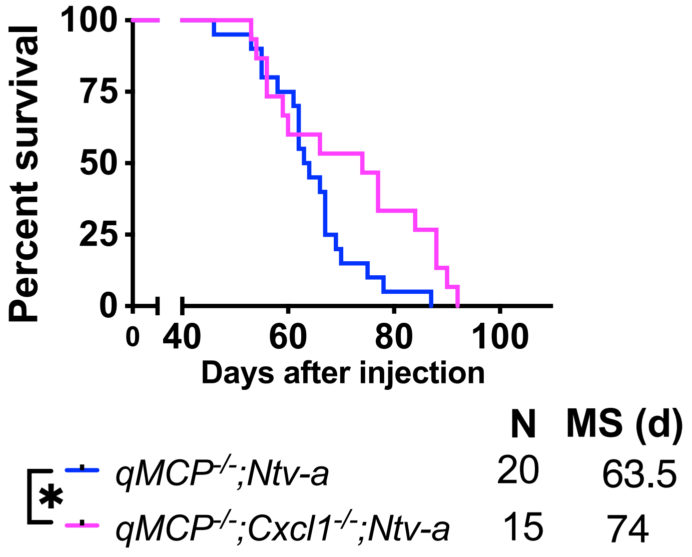

Supplementary Figure 31. Kaplan-Meier survival curves of *qMCP$^{-/-}$; Ntv-a* and *qMCP$^{-/-}$; Cxcl1$^{-/-}$;Ntv-a* mice bearing Nf-1 sliced tumors. Log-rank test. P = 0.0424. MS = median survival, N = number of mice. Source data are provided as a Source Data file.

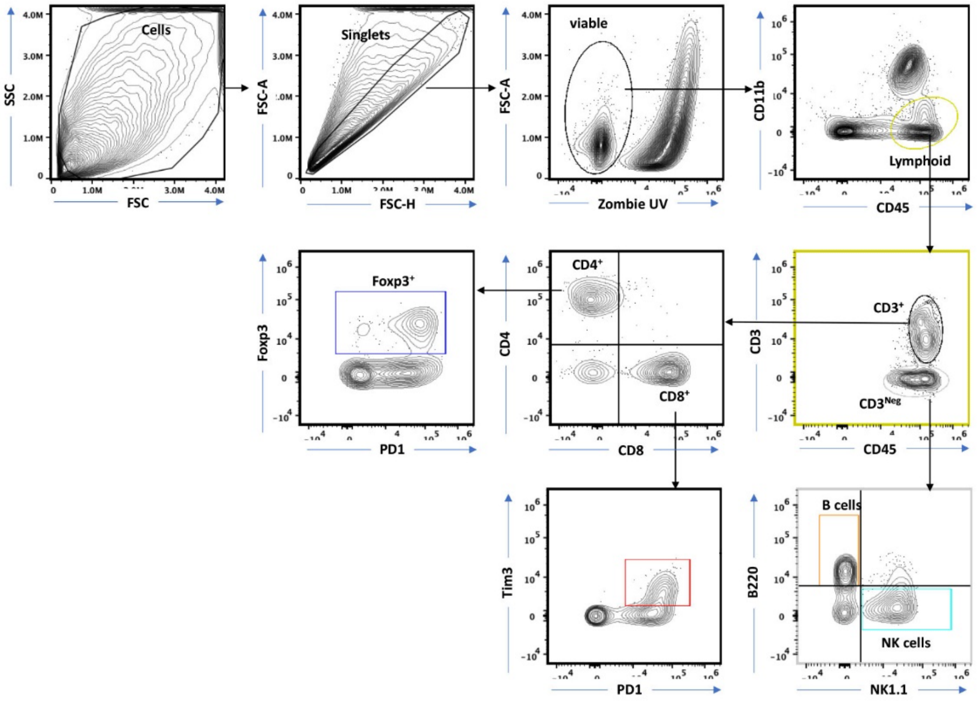

Supplementary Figure 32. Spectral flow cytometry analysis of lymphoid cells in HCC. Gating strategy is shown.

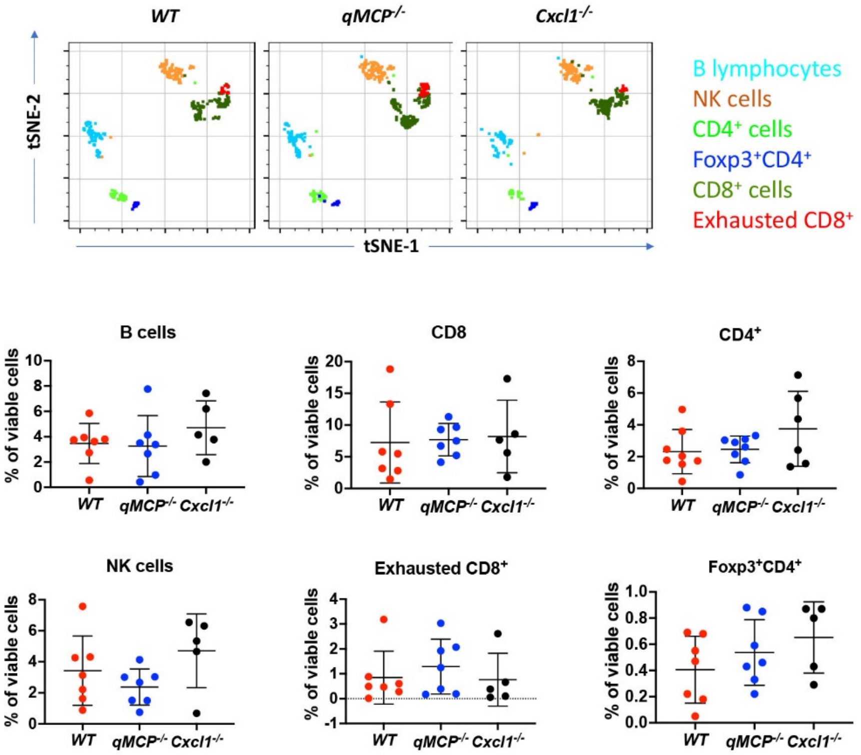

Supplementary Figure 33. Spectral flow cytometry analysis of lymphoid cells in HCC. (A) tSNE plots illustrating lymphoid subsets of immune cells in HCC. (B) Quantification of lymphoid cells in HCC by spectral flow cytometry. N = 7 (*WT*)*,* *7* (*qMCP$^{-/-}$*), and 5 (*Cxcl1$^{-/-}$)* mice. Data are presented as mean +/- SD. Source data are provided as a Source Data file.

Supplementary Table 1. Patient information.

Sample #	Age	Sub-classification	Molecular driver
1	74	MES	NF1 mutation
2	66	CL	EGFR amplified, TERT promoter c-146C>T, TP53 p.Y220D missense; PTEN loss; CDKN2A/B loss
3	46	CL	EGFR amplified; EGFR G598V - subclonal, EGFRvIVa† (ex 24-27 del); ATM splice site 3576G>A; KDR R961W; CDKN2A/B loss; MTAP loss; TERT promoter -124C>T; VUS: CARD11 A687V; CEBPA V287G; CRKL T213A; GATA4 R318T; MAP3K1 T1511I; MTAP M169V; NTRK1 G18E; NTRK2 S167Y; PTEN F154I; SETD2 V932I;
4	56	MES	NF1 splice site 2850+1G>A; CDKN2A loss; PTEN M199del; TERT -146C>T; TP53 T211_H214del; MS-Stable; 4 muts/ MB
5	58	CL	EGFR amplified; EGFR p.A289V; PTEN p.K332Tfs*8; TERT c.-124C>T
6	75	MES	CDKN2A loss; MTAP loss; NF1 N2387_F2388de; NF1 K1444E; PTEN loss; PTPN11 Y63C; TERT promoter -146C>T; MS-Stable; 1 mut/Mb;
7	74	PN	PDGFRa and KIT amplifications

Supplementary Table 2. qPCR Primers Used in Study. The Bio-Rad qPCR primers used in the study are listed with their catalog numbers.

**Primer**	**Bio-Rad Catalog Number**
Abcg2	qMmuCID0009104
Actb	qMmuCED0027505
Aif1	qMmuCED0046745
Ascl1	qMmuCED0044820
Casp1	qMmuCID0026983
Ccl11	qMmuCED0044849
Ccl12	qMmuCED0061017
Ccl2	qMmuCED0048300
Ccl7	qMmuCED0049027
Ccl8	qMmuCED0003781
Ccr1	qMmuCID0006862
Cd44	qMmuCID0025677
Cebpb	qMmuCED0050360
Chi3l1	qMmuCID0015758
Cxcl3	qMmuCED0001059
Cxcl5	qMmuCED0003886
Dll3	qMmuCID0023659
Gfap	qMmuCID0020163
Hprt	qMmuCED0045738
Met	qMmuCID0017026
Mgmt	qMmuCID0009593
Ncam1	qMmuCID0005870
Olig2	qMmuCED0003760
Rps6ka3	qMmuCID0006067
Serpine1	qMmuCID0027303
Sox2	qMmuCED0051857
Stat3	qMmuCED0044698
Taz	qMmuCID0020469
Tgfb1	qMmuCID0017320
Yap1	qMmuCID0005990

Supplementary Table 3. Primary antibodies used in the study.

Antibody	Application	Specificity	Manufacturer	Dilution	Catalog Number
IBA1	IHC/IF	Human, Mouse	Wako	1:1500	019-19741
Anti-Ly6g	In vivo injection	Mouse	BioXcell	1 mg/ml	BE0075-1
Anti-trinitrophenol	In vivo injection	Mouse	BioXcell	1 mg/ml	BE0089
OLIG2	IHC	Mouse	Millipore Sigma	1:500	AB9610
CD31	IHC	Mouse	Dianova	1:50	DIA-310
CD44	IHC	Human, Mouse	BD Pharmingen	1:100	550538
GFAP	IHC	Mouse	CST	1:10000	3670
Elane	IHC	Human, Mouse	Bioss	1:400	bs6982R
Elane	IHC	Human, Mouse	Abcam	1:400	ab68672
P_2Y_{12}	IHC	Mouse	AnaSpect	1:500	SQ-ANAB-78839
TMEM119	IHC	Mouse	Abcam	1:100	Ab209064
CD45-APC	Flow Cytometry	Mouse	BioLegend	1:100	103112
CD45-PE	Flow Cytometry	Mouse	BioLegend	1:100	103106
CD45-FITC	Flow Cytometry	Mouse	BioLegend	1:100	103107
CD45-V450	Flow Cytometry	Mouse	BD Biosciences	1:100	560501
CD45-BV510	Flow Cytometry	Mouse	BD Biosciences	1:100	563891
B220-BV605	Flow Cytometry	Mouse	BioLegend	1:100	103243
B220-AF700	Flow Cytometry	Mouse	BioLegend	1:100	103232
CD101-APC	Flow Cytometry	Mouse	Invitrogen	1:100	17101180
CD103-BUV395	Flow Cytometry	Mouse	BD Biosciences	1:100	748253
CD11b-APC-Cy7	Flow Cytometry	Mouse	BioLegend	1:100	101226
CD11b-PerCP-Cy5.5	Flow Cytometry	Mouse	BD Biosciences	1:100	550993
CD11c-PE-Dazzle594	Flow Cytometry	Mouse	BioLegend	1:100	117348
CD11c-APC	Flow Cytometry	Mouse	BD Biosciences	1:100	550281
CD19-BV785	Flow Cytometry	Mouse	BD Biosciences	1:100	563333
CD24-BUV496	Flow Cytometry	Mouse	BD Biosciences	1:200	612953
CD3-PE-dazzle	Flow Cytometry	Mouse	BioLegend	1:100	100348
CD4-APC-Cy7	Flow Cytometry	Mouse	BioLegend	1:100	100526
CD49d-PE-dazzle	Flow Cytometry	Mouse	BioLegend	1:100	103625
CD5-PE-Cy5	Flow Cytometry	Mouse	BioLegend	1:100	100610
CD8-BV510	Flow Cytometry	Mouse	BioLegend	1:100	100752
CX3CR1-PerCP-Cy5.5	Flow Cytometry	Mouse	BioLegend	1:100	149009
CX3CR1-BV650	Flow Cytometry	Mouse	BioLegend	1:100	149033
CXCR2-PE	Flow Cytometry	Mouse	BioLegend	1:100	149609
F4/80-PE-Cy7	Flow Cytometry	Mouse	BioLegend	1:100	123114

F4/80-BV711	Flow Cytometry	Mouse	BioLegend	1:100	123147
Foxp3-FITC	Flow Cytometry	Mouse	Invitrogen	1:50	11-5773-82
Gr-1-BV711	Flow Cytometry	Mouse	BioLegend	1:100	108443
GrzmB-PE	Flow Cytometry	Human, Mouse	Invitrogen	1:50	12-8899-41
IA/IE-BV650	Flow Cytometry	Mouse	BD Biosciences	1:100	563415
IA/IE-Alex700	Flow Cytometry	Mouse	BioLegend	1:100	107622
Ly6c-AF488	Flow Cytometry	Mouse	BioLegend	1:200	128022
Ly6c-PE-Cy7	Flow Cytometry	Mouse	BD Biosciences	1:200	560593
Ly6g-V450	Flow Cytometry	Mouse	BD Biosciences	1:100	560603
NK1.1-AF647	Flow Cytometry	Mouse	BioLegend	1:100	108720
NK1.1-BV711	Flow Cytometry	Mouse	BD Biosciences	1:100	740663
PD-L1-BV605	Flow Cytometry	Mouse	BD Biosciences	1:100	745135
PD1-BV785	Flow Cytometry	Mouse	BioLegend	1:100	135225
Tim-3-PE-Cy7	Flow Cytometry	Mouse	Invitrogen	1:100	25-5870-82
