# Supplementary material for: Monocyte depletion enhances neutrophil influx and proneural to mesenchymal transition in glioblastoma
Source: Nat Commun. 2023 Apr 3;14:1839. doi: 10.1038/s41467-023-37361-8 (PMC10070461; doi:10.1038/s41467-023-37361-8)
Supplement: Supplementary file 3 — Reporting Summary [file 41467_2023_37361_MOESM3_ESM.pdf]

nature portfolio

Corresponding author(s): Zhihong Chen, Dolores Hambardzumyan

Last updated by author(s): Feb 22, 2023

Reporting Summary

Nature Portfolio wishes to improve the reproducibility of the work that we publish. This form provides structure for consistency and transparency in reporting. For further information on Nature Portfolio policies, see our Editorial Policies and the Editorial Policy Checklist.

Statistics

For all statistical analyses, confirm that the following items are present in the figure legend, table legend, main text, or Methods section.

n/a	Confirmed	
☐	☒	The exact sample size (*n*) for each experimental group/condition, given as a discrete number and unit of measurement
☐	☒	A statement on whether measurements were taken from distinct samples or whether the same sample was measured repeatedly
☐	☒	The statistical test(s) used AND whether they are one- or two-sided<br>*Only common tests should be described solely by name; describe more complex techniques in the Methods section.*
☒	☐	A description of all covariates tested
☒	☐	A description of any assumptions or corrections, such as tests of normality and adjustment for multiple comparisons
☐	☒	A full description of the statistical parameters including central tendency (e.g. means) or other basic estimates (e.g. regression coefficient) AND variation (e.g. standard deviation) or associated estimates of uncertainty (e.g. confidence intervals)
☐	☒	For null hypothesis testing, the test statistic (e.g. *F*, *t*, *r*) with confidence intervals, effect sizes, degrees of freedom and *P* value noted<br>*Give P values as exact values whenever suitable.*
☒	☐	For Bayesian analysis, information on the choice of priors and Markov chain Monte Carlo settings
☒	☐	For hierarchical and complex designs, identification of the appropriate level for tests and full reporting of outcomes
☒	☐	Estimates of effect sizes (e.g. Cohen's *d*, Pearson's *r*), indicating how they were calculated

Our web collection on statistics for biologists contains articles on many of the points above.

Software and code

Policy information about availability of computer code

Data collection	IVY Glioblastoma Atlas Project database (https://glioblastoma.alleninstitute.org/) was searched for spatial expression of MCP genes; TCGA (U133 Microarray data for the GBM provisional) dataset was searched for subtype-specific information of survival, age, gender and selected gene expressions; NDP.view2 software (Hamamatsu) was used to view and collect IHC images; BD Diva and Cytek SpectroFlo were used to collected flow cytometry data. Cell Ranger v5.0.0 (lOX Genomics) was used to align single cell RNA seq data to mouse genome reference mm10 customized to include Rfp sequence.
Data analysis	NIH imaging analysis software Fiji (RRID:SCR_002285) was used for imaging analysis; Matlab (Mathworks) was used to generate a PCA graph; Morpheus matrix visualization tool (Broad Institute) was used for matrix analysis of proteomics results; NanoString Nsolver software was used to visualize and analyze NanoString data; Graphpad Prism was used for statistical analyses; FlowJo 10 software (Tree Star Inc., RRID:SCR_008520) was used for flow data analyses; Single-cell RNA-seq data was analyzed using R v4.2.2 with the following packages: Seurat v4.0.5, Harmony v1.0, scWGCNA v0.2.11, clusterProfiler v4.2.0, CellPhoneDB v2.0.

For manuscripts utilizing custom algorithms or software that are central to the research but not yet described in published literature, software must be made available to editors and reviewers. We strongly encourage code deposition in a community repository (e.g. GitHub). See the Nature Portfolio guidelines for submitting code & software for further information.

Data

Policy information about availability of data

All manuscripts must include a data availability statement. This statement should provide the following information, where applicable:

- Accession codes, unique identifiers, or web links for publicly available datasets
- A description of any restrictions on data availability
- For clinical datasets or third party data, please ensure that the statement adheres to our policy

The data that support the findings of this study are available from the accompanied “Source Data file”. ScRNA-Seq data were deposited at GEO with accession number GSE203154 [https://www.ncbi.nlm.nih.gov/geo/query/acc.cgi?acc=GSE203154]. Newly created qMCP-KO mice will be distributed to interested colleagues upon mutually satisfactory materials transfer agreements. Source data are provided with this paper. The remaining data are available within the Article, Supplementary Information or Source Data file.

Human research participants

Policy information about studies involving human research participants and Sex and Gender in Research.

Reporting on sex and gender	Specimen from both sexes were used in this study.
Population characteristics	Population characteristics were summarized in Supplemental Table 1. Age, sex and GBM diagnosis and genetic drivers were described in the table.
Recruitment	Only archived specimen were used in this study obtained from Mount Sinai biorepository under IRB approved protocols. No prospective recruitment of patients were performed.
Ethics oversight	Fresh tumor tissues were collected at Mount Sinai Hospital through the biorepository, under IRB-approved protocols (18-00983).

Note that full information on the approval of the study protocol must also be provided in the manuscript.

Field-specific reporting

Please select the one below that is the best fit for your research. If you are not sure, read the appropriate sections before making your selection.

☒ Life sciences ☐ Behavioural & social sciences ☐ Ecological, evolutionary & environmental sciences

For a reference copy of the document with all sections, see nature.com/documents/nr-reporting-summary-flat.pdf

Life sciences study design

All studies must disclose on these points even when the disclosure is negative.

Sample size	Sample size was determined based on similar studies previously performed and published by the lab. Power tests were performed with known mean values and standard deviation that are relatable to experiments described here. The alpha value set at 0.05 and desired power at 0.80.
Data exclusions	Data exclusion were performed with Grubb's test with the alpha set at 0.05.
Replication	All experiments were reproduced to reliably support conclusions stated in the manuscript. Specifically, survival analyses was performed at least twice with minimum 8 mice per group included each time. All IHC images presented were imaged at least 5 independent regions for a single data point. For flow cytometry analysis, at least five independent biological replicates were used for each genotype. For scRNA-seq analysis, three independent biological replicates were used for each genotype. RNAscope, qPCR and in vitro culture experiments were repeated at least two times, with consistent results.
Randomization	Animals were randomly divided into experimental groups while assuring gender balance.
Blinding	The experimentalists were blinded of the genotypes during data collection and when analyzing the data.

Reporting for specific materials, systems and methods

We require information from authors about some types of materials, experimental systems and methods used in many studies. Here, indicate whether each material, system or method listed is relevant to your study. If you are not sure if a list item applies to your research, read the appropriate section before selecting a response.

Materials & experimental systems

n/a	Involved in the study	
	x	Antibodies
	x	Eukaryotic cell lines
x		Palaeontology and archaeology
	x	Animals and other organisms
	x	Clinical data
x		Dual use research of concern

Methods

n/a	Involved in the study	
x		ChIP-seq
	x	Flow cytometry
x		MRI-based neuroimaging

Antibodies

Antibodies used

1, IBA1, Wako, Cat# 019-19741
2, Anti-Ly6g, BioXcell, Cat# BE0075-1
3, Anti-trinitrophenol, BioXcell, Cat# BE0089
4, OLIG2, Millipore Sigma, Cat# AB9610
5, CD31, Dianova, Cat# DIA-310
6, CD44, BD Pharmingen, Cat# 550538
7, GFAP, CST, Cat# 3670
8, Elane, Bioss, Cat# bs6982R
9, Elane, AbCam, Cat# ab68672
10, P2Y12, AnaSpect, Cat# SQ-ANAB-78839
11, CD45-APC, BioLegend, Cat# 103112
12, CD45-PE, BioLegend, Cat# 103106
13, CD45-FITC, BioLegend, Cat# 103107
14, CD45-V450, BD Biosciences, Cat# 560501
15, CD45-BV510, BD Biosciences, Cat# 563891
16, B220-BV605, BioLegend, Cat# 103243
17, B220-AF700, BioLegend, Cat# 103232
18, CD101-APC, Invitrogen, Cat# 17101180
19, CD103-BUV395, BD Biosciences, Cat# 748253
20, CD11b-APC-Cy7, BioLegend, Cat# 101226
21, CD11b-PerCP-Cy5.5, BD Biosciences, Cat# 550993
22, CD11c-PE-Dazzle594, BioLegend, Cat# 117348
23, CD11c-APC, BD Biosciences, Cat# 550281
24, CD19-BV785, BD Biosciences, Cat# 563333
25, CD24-BUV496, BD Biosciences, Cat# 612953
26, CD3-PE-dazzle, BioLegend, Cat# 100348
27, CD4-APC-Cy7, BioLegend, Cat# 100526
28, CD49d-PE-dazzle, BioLegend, Cat# 103625
29, CD5-PE-Cy5, BioLegend, Cat# 100610
30, CD8-BV510, BioLegend, Cat# 100752
31, CX3CR1-PerCP-Cy5.5, BioLegend, Cat# 149009
32, CX3CR1-BV650, BioLegend, Cat# 149033
33, CXCR2-PE, BioLegend, Cat# 149609
34, F4/80-PE-Cy7, BioLegend, Cat# 123114
35, F4/80-BV711, BioLegend, Cat# 123147
36, Foxp3-FITC, Invitrogen, Cat# 11-5773-82
37, Gr-1-BV711, BioLegend, Cat# 108443
38, GrzmB-PE, Invitrogen, Cat# 12-8899-41
39, IA/IE-BV650, BD Biosciences, Cat# 563415
40, IA/IE-Alex700, BioLegend, Cat# 107622
41, Ly6c-AF488, BioLegend, Cat# 128022
42, Ly6c-PE-Cy7, BD Biosciences, Cat# 560593
43, Ly6g-V450, BD Biosciences, Cat# 560603
44, NK1.1-AF647, BioLegend, Cat# 108720
45, NK1.1-BV711, BD Biosciences, Cat# 740663
46, PD-L1-BV605, BD Biosciences, Cat# 745135
47, PD1-BV785, BioLegend, Cat# 135225
48, Tim-3-PE-Cy7, Invitrogen, Cat# 25-5870-82

Validation

All antibodies were freshly obtained from the manufacturer which provided certificates of analysis as inserts.

Eukaryotic cell lines

Policy information about cell lines and Sex and Gender in Research

Cell line source(s)	DF-1 cells from ATCC
Authentication	DF-1 cells were purchased from ATCC which has assurance in place. These cells were immediately used for experiments
Mycoplasma contamination	Mycoplasma was routinely tested at three month intervals to affirm no maycoplasma contamination occurs in our cell cultures.
Commonly misidentified lines (See ICLAC register)	No commonly misidentified cell types were used in this study.

Animals and other research organisms

Policy information about studies involving animals; ARRIVE guidelines recommended for reporting animal research, and Sex and Gender in Research

Laboratory animals	Mice of both sexes (equal distribution) in the age range of 6-12 weeks were used for experiments. Previously-described Ccl2-/- (Jackson laboratory, #004434), Ccl7-/- (Jackson laboratory, #017638), Ccl8/12-/- (gifted by Dr. Sabina Islam), Cxcll-/- (Shea-Donohue et al., 2008), and Cxcr2-/- (Jackson laboratory, #006848) mice were either maintained as single knock-out strains, or cross-bred to the Ntv-a mice to generate double or triple knock-out strains. Cx3Cr1GFP/WT;Ccr2RFP/RFP and Cx3Cr1GFP/WT;Ccr2RFP/RFP mice were generated from heterozygous breeding pairs (Jackson laboratory #005582 and #017586), backcrossed for more than 10 generations. All these mice are in a C57BL/6 background. C57BL/6J mice (#000664) at 6 weeks old were purchased from the Jackson labs. All animals were housed in a climate-controlled (18-23 oC and 40-60% humidity), pathogen-free facility with access to food and water ad libitum under a 12-hour light/dark cycle.
Wild animals	This study does not involve any wild animals.
Reporting on sex	Both sexes were used in this study.
Field-collected samples	This study does not involve any Field-collected samples.
Ethics oversight	All experimental procedures were approved by the Institutional Animal Care and Use Committee (IACUC) of Emory University (Protocol #2017-00633) and the Icahn School of Medicine at Mount Sinai (Protocol #2019-00619 and #2014-0229).

Note that full information on the approval of the study protocol must also be provided in the manuscript.

Clinical data

Policy information about clinical studies

All manuscripts should comply with the ICMJEguidelines for publication of clinical research and a completedCONSORT checklist must be included with all submissions.

Clinical trial registration	No Clinical trial was used in this study.
Study protocol	No Clinical trial was used in this study, therefore, no protocol is used.
Data collection	No Clinical data was collected in this study.
Outcomes	No Clinical trial was used in this study.

Flow Cytometry

Plots

Confirm that:

- [x] The axis labels state the marker and fluorochrome used (e.g. CD4-FITC).
- [x] The axis scales are clearly visible. Include numbers along axes only for bottom left plot of group (a 'group' is an analysis of identical markers).
- [ ] All plots are contour plots with outliers or pseudocolor plots.
- [x] A numerical value for number of cells or percentage (with statistics) is provided.

Methodology

Sample preparation	Mice were sacrificed at humane endpoint with an overdose of ketamine and xylazine and perfused with 10 ml cold Ringer's solution. The brain was carefully extracted, tumors were dissected from the brain, minced into pieces < 1 mm3, and digested with an enzymatic mixture of 0.5% collagenase D (Sigma, 11088858001) and DNase I (Roche, 11284932001). Single-cell suspensions were passed through 70 µm cell strainers, centrifuged, and resuspended in 30% Percoll (GE Healthcare,

17-0891-01) solution containing 10% FBS (Hyclone SH30396.03). Cells were separated by centrifugation at 800g for 15 minutes at 4°C. The supernatant was carefully removed to discard debris and lipids. The cells were then washed in cold PBS and resuspended in RBC lysis buffer (BioLegend, 420301) for 1 min at 37°C. Cells were transferred to an Eppendorf tube and washed once with FACS buffer (DPBS with 0.5% BSA) and blocked with 100 µl of 2x blocking solution (2% FBS, 5% normal rat serum, 5% normal mouse serum, 5% normal rabbit serum, 10 µg/ml anti-FcR (BioLegend, 101319) and 0.2% NaN3 in DPBS) on ice for 30 minutes. Cells were then stained with primary antibodies (Table S2) on ice for 30 minutes and washed with PBS. The cells were subsequently incubated in 100 µl viability dye (Zombie UV, BioLegend, 1:800) at room temperature for 20 min. The cells were washed and fixed with fixation buffer (eBioscience, 00-5123-43, 00-5223-56) for 30 min at 4 °C. Cells were washed and stained with the cocktail of antibodies examined myeloid lineage are set aside in the fridge until loading to the cytometer. Cells stained for the lymphoid panel were then permeabilized with a permeabilization buffer (eBioscience, 00-8333-56) before the intracellular markers were stained. The cells were washed and stored in fridge till analysis.

Instrument

BD Symphony, Fortessa II and Cytek Aurora were used for data collection.

Software

BD Diva and Cytek SpectroFlo were used for data collection.

Cell population abundance

Sorting was not used in this study.

Gating strategy

Gating strategies were illustrated in supplementary figures. all analysis starts with FSC/SSC plots, followed by doublet exclusion and viability inclusion.

☒ Tick this box to confirm that a figure exemplifying the gating strategy is provided in the Supplementary Information.
